# Indicators and Recommendations for Assessing Sustainable Healthy Diets

**DOI:** 10.3390/foods10050999

**Published:** 2021-05-02

**Authors:** Maite M. Aldaya, Francisco C. Ibañez, Paula Domínguez-Lacueva, María Teresa Murillo-Arbizu, Mar Rubio-Varas, Beatriz Soret, María José Beriain

**Affiliations:** 1 Institute on Innovation & Sustainable Development in the Food Chain (IS-FOOD), Public University of Navarra (UPNA), Jerónimo de Ayanz Building, Arrosadia Campus, 31006 Pamplona, Spain; pi@unavarra.es (F.C.I.); mariateresa.murillo@unavarra.es (M.T.M.-A.); soret@unavarra.es (B.S.); mjberiain@unavarra.es (M.J.B.); 2 School of Sciences, University of Navarra, 31080 Pamplona, Spain; pdominguez.7@alumni.unav.es; 3 Institute for Advanced Research in Business and Economics (INARBE), Public University of Navarra (UPNA), Jerónimo de Ayanz Building, Arrosadia Campus, 31006 Pamplona, Spain; mar.rubio@unavarra.es

**Keywords:** sustainable healthy diet, food environmental sustainability, socioeconomic sustainability, indicators, constraints, costs

## Abstract

Research coupling human nutrition and sustainability concerns is a rapidly developing field, which is essential to guide governments’ policies. This critical and comprehensive review analyzes indicators and approaches to “sustainable healthy diets” published in the literature since this discipline’s emergence a few years ago, identifying robust gauges and highlighting the flaws of the most commonly used models. The reviewed studies largely focus on one or two domains such as greenhouse gas emissions or water use, while overlooking potential impact shifts to other sectors or resources. The present study covers a comprehensive set of indicators from the health, environmental and socio-economic viewpoints. This assessment concludes that in order to identify the best food option in sustainability assessments and nutrition analysis of diets, some aspects such as the classification and disaggregation of food groups, the impacts of the rates of local food consumption and seasonality, preservation methods, agrobiodiversity and organic food and different production systems, together with consequences for low-income countries, require further analysis and consideration.

## 1. Introduction

Environmental degradation and malnutrition, in all its forms, are both occurring at an accelerated pace around the world. While the causes are complex, unhealthy diets coupled with unsustainable food systems can be considered among the main contributors to these global burdens [1].

Referring to environmental sustainability, currently, the global food system is the largest freshwater user: agriculture alone accounts for 70% of freshwater withdrawn in the world [2]. Agriculture is also responsible for 21–37% of total greenhouse gas (GHG) emissions [3] and covers approximately 49–51% of global ice-free land surface, with grazing land representing 37% and croplands representing approximately 12–14% [4]. Intensive and unsustainable agricultural practices and pollution can also trigger biodiversity loss [5].

In regard to the health component, currently, an estimated 821 million people are undernourished, 151 million children under five years of age are stunted, 613 million women and girls aged 15 to 49 suffer from iron deficiency, and, on the other side, 2 billion adults are overweight or obese [3]. Nowadays, unhealthy and unbalanced diets pose an increased risk to morbidity and mortality.

The challenge of achieving healthy diets is coupled with the challenge of attaining sustainable food systems [6]. While food production contributes to natural resource depletion and diets should improve to overcome malnutrition, sustainable food consumption and production could also be considered an opportunity for enhancing human health and environmental sustainability.

In 2011, Riley and Buttriss raised the question on “which dietary patterns are both healthy and sustainable?”, although they were not able to provide a complete answer due to the complexity of the issue [7]. Given the divergence of approaches, in 2019, the FAO and WHO held a consultation and coined the concept “sustainable healthy diets”. This was defined as:

*“dietary patterns that promote all dimensions of individuals’ health and wellbeing; have low environmental pressure and impact; are accessible, affordable, safe and equitable; and are culturally acceptable”* [1]

Sustainable healthy diets must combine all the dimensions of sustainability to avoid unintended consequences. However, currently, a few dietary guidelines take environmental sustainability into account, such as those of the Netherlands [8], Nordic countries [9], Germany [10], Brazil [11], Sweden [12], Qatar [13] and France [14]. Furthermore, the papers published in the literature generally focus on specific aspects of health, environmental or socioeconomic sustainability, sometimes leaving out one or two of the three components. Further development of encompassing indicators and data on all dimensions of sustainability is needed to make this concept complete, useful and effective.

In recent years, there has been an increase in the number of systematic reviews focused on sustainable and healthy diets, most of which also have a specific scope. For instance, some of the reviews have a limited geographical reach, focusing on one country such as the UK [15] or the USA [16]. Other reviews focus on a specific domain such as mathematical optimization studies [17] or labeling schemes [18]. Most reviews have a specific environmental scope, analyzing a single environmental aspect [19,20] or two or three environmental resources [15,21,22]. Some leave socioeconomic aspects out of the scope of review, instead focusing on the interlinkages between the environment and diets [23,24]. Few reviews combine socioeconomic and environmental performance with nutritional and health indicators [17,25,26], and only three of these compile [27] and recommend [28,29] criteria. There has been no comprehensive review highlighting a complete set of indicators coupled with an analysis of the gaps of knowledge and misconceptions from a multidisciplinary perspective. Thus, limited evidence is available on the trade-offs involved in selecting sustainable healthy diets.

The current critical review paper aims to identify a comprehensive set of indicators for assessing sustainable healthy diets, analyzing the most common shortcomings from a health, environmental and socio-economic perspective. First, a literature search is performed to identify frequently used indicators and approaches in these three domains. Second, a section is devoted to outlining some of the gaps in knowledge and frequent misconceptions around sustainable healthy diets. Third, a comprehensive collection of interdisciplinary indicators is provided, proposing, among other actions, further research on the classification of food groups, impacts of different production systems and consequences for low-income countries to develop a complete understanding for decision-making.

## 2. Materials and Methods

**Figure 1:**
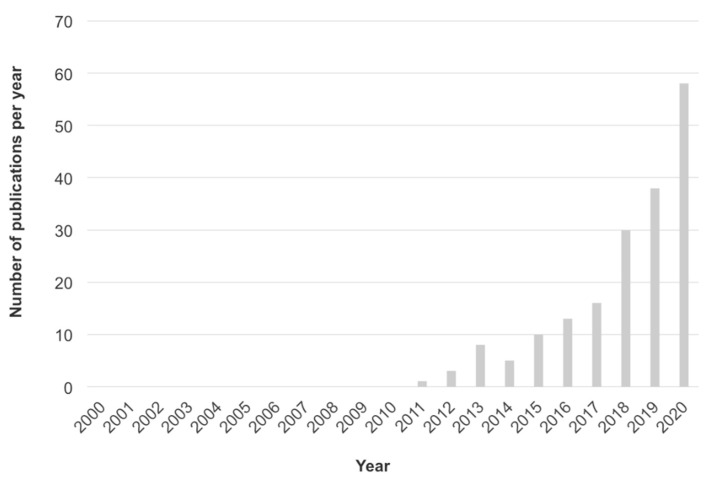
Trend in the number of publications using the search terms “healthy and sustainable diet”, “sustainable and healthy diet”, “sustainable diet” and “healthy diet”, “sustainable healthy diet”, “healthy sustainable diet” published from 2000–2020, excluding 2021, and listed on Scopus, Web of Science and PubMed (28 February 2021).

**Figure 2:**
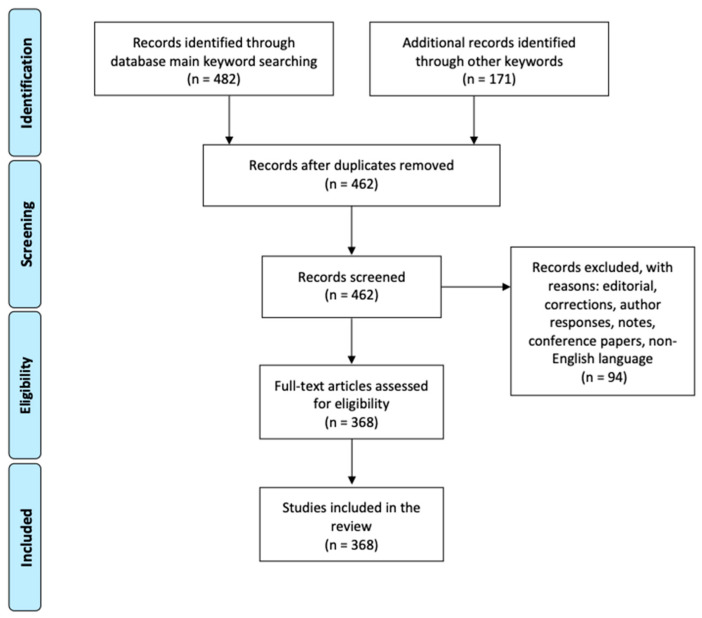
PRISMA flow diagram of the selection process of indicators for assessing research papers on sustainable healthy diets. Main keywords: “healthy and sustainable diet”, “sustainable and healthy diet”, “sustainable diet” and “healthy diet”, “sustainable healthy diet”, “healthy sustainable diet”. Other keywords: see Table 1 (28 February 2021).

The literature search was carried out using three bibliographical databases: Scopus, Web of Science and PubMed. The search was limited to items written in English between 2000 and 28 February 2021. Editorials, corrections, author responses, notes, and conference papers were discarded. An initial screening was performed using the keywords “healthy and sustainable diet”, “sustainable and healthy diet”, “sustainable diet” and “healthy diet”, “sustainable healthy diet”, and “healthy sustainable diet”. Figure 1 shows the results of this screening. Records identified through these main keyword search amounted to 197,482 if including duplicates and exclusions. Additionally, other keywords were used for deepening in specific indicators (Table 1), identifying 171 additional records. Each of these keywords was combined with the “sustainable diet” term. In addition, the filters “review”, “systematic review”, “meta-analysis”, and “human species” were used for the domain “nutrition and health”. The abstracts were first reviewed before moving to the full text. Two researchers (M.M.A. and F.C.I.) reviewed the papers and coordinated with the different field experts (M.R.-V., B.S., M.T.M.-A. and P.D.). Any discrepancies were resolved by consulting a third reviewer (M.J.B.). Articles with no clearly identifiable indicators or approaches for assessing sustainable healthy diets or food systems were excluded. Selected articles were de-duplicated by a search tool in Zotero citing manager [30] (Figure 2).

## 3. Healthy Sustainable Diets in the Literature

### 3.1. Healthy Diet

Some of the latest studies point to the following dietary recommendations in promoting overall wellbeing and low risk of major chronic disease: (1) protein sources primarily from plants, including soy foods; other legumes; and nuts, fish or alternative sources of n-3 polyunsaturated fatty acids (PUFA) consumed several times per week with optional modest consumption of poultry and eggs and low intakes of red meat, if any, and especially of processed meat; (2) fat obtained mostly from unsaturated plant sources with low intakes of saturated fats and no consumption of partly hydrogenated oils; (3) carbohydrates primarily from whole grains with low intake of refined grains and less than 5% of energy from sugar; (4) at least five daily servings of fresh fruits and non-starchy vegetables; and (5) optional moderate dairy consumption [6,31,32,33]. These components can be combined in various types of omnivore, vegetarian, and vegan diets [6]. This nutritional guidance improves the intake of most nutrients. However, specific cases of dietary inadequacies require obtaining nutrients from dietary supplements or enriched foods [34,35,36,37,38,39,40,41]. The most accepted nutritional criteria proposed for a healthy diet are summarized in Table 2.

Some studies analyzing the association between health and diet are based on preconceived concepts and established hypotheses that do not support the cause–effect results and do not take into consideration the sustainability of the assessed diets. A balanced and healthy diet should be based on available, accessible, affordable, safe and culturally acceptable food and allow guaranteeing socio-economic and environmental sustainability.

### 3.2. Environmentally Sustainable Diet

The two main approaches used to address the environmental sustainability of diets and food systems are life cycle analysis (LCA) and environmental footprints. LCA assesses the environmental impact of a product from resource extraction, manufacturing, and transport to use and end-of-life disposal [143]. Ideally, LCA studies cover every relevant environmental category. However, in the case of diet-related impact assessments, only a few environmental indicators are generally used to perform analyses. The most common and recurrent impact categories applied in these studies are climate change, freshwater use, land use, acidification, ecotoxicity, eutrophication, human toxicity, ionizing radiation, ozone depletion, particulate matter, photochemical ozone formation and resource depletion [144,145,146,147,148,149,150]. There are no standardized methodologies to perform LCAs for diets. Thus, authors add and discriminate environmental indicators in different ways, leading to a wide variety of studies that differ in scale and sets of environmental indicators, hindering data comparisons.

Environmental footprint approaches are able to pair food-production estimates with country-specific environmental footprints and compare them with planetary boundaries [151,152]. The footprint indicators used in sustainable diet studies are GHG emissions, freshwater use, land use and nitrogen, phosphorus application, biodiversity, energy and the ecological footprint [153,154,155,156,157,158,159,160,161]. However, many authors do not adopt these methodologies from a holistic perspective to assess the environmental impact from diets. The vast majority of studies take into consideration a single or few environmental aspects or impact categories (Table 3). Therefore, the results obtained from these kinds of assessments have to be interpreted rigorously as they may show a reductionist outlook of the whole environmental impact.

Recommendations from wealthier countries such as Europe include reducing the consumption of certain products, such as red meat and sugar, particularly by reducing excessive consumption, and increasing the consumption of fruits, vegetables, nuts and legumes [6,162]. Beyond these relevant global trends, a deeper understanding of the impacts of different production systems would be useful to improve and facilitate the decision-making. Furthermore, these methodologies do not generally consider aspects such as the rate of local/regional food consumption and seasonality, agrobiodiversity and organic/eco-friendly production and consumption [163]. These approaches are discussed in detail in Section 4.2.

### 3.3. Socioeconomic Approach to a Sustainable Healthy Diet

Food security remains the most significant challenge to the development of sustainable and healthy diets. Over 2 billion people, mostly in low- and middle-income countries, do not have regular access to safe, nutritious and sufficient food [217]. However, irregular access is also a challenge for high-income countries, including for 8% of the populations of North America and Europe. Most environmental studies on sustainable diets neglect or minimize socioeconomic factors, rendering their recommendations empirically unfeasible. Furthermore, there is a bias in the geographical focus of studies towards high- and middle-income countries. Of the country-specific studies analyzed, 121 address high-/middle-income countries, while only 26 focus on low-income countries. Dietary choices have macroeconomic and microeconomic implications for both the producer (supply) and consumer (demand) sides. Most studies identify criteria affecting consumer behavior—either affordability and/or acceptability (Table 4 and Table S3). A small number of studies consider the distinct constraints that food producers face when adopting the production of healthy food and using methods that minimize environmental damage. Another strand of literature analyzes the value chains that take products from suppliers to the consumer. What is missing from the literature are comprehensive socioeconomic approaches based on criteria that affect supply and demand and the necessary value chains that connect them.

## 4. Dispelling the Misconceptions

### 4.1. Nutrition and Health Approaches

The WHO establishes that a healthy diet protects against malnutrition and forms the basis for health and development, preventing the development of diseases including diabetes, cardiovascular diseases, some cancers and other conditions linked to obesity [234]. However, defining a global healthy diet is a challenge given the multiple factors to consider as people have different nutritional needs according to their age, sex, disease status and physical activity levels, and the particularities of vulnerable populations, such as young children and pregnant women, must also be taken into account [6]. In line with the WHO, in some countries and social classes the excessive consumption of low nutritional quality foods, which are generally inexpensive, and of certain animal products among some countries and social classes, can lead to excessive sugar, salt, fat and energy intakes. This section brings to light several concepts mentioned in Table 2 of this work that are sometimes unclear and considered important to achieve a balanced and high-quality nutrition.

#### 4.1.1. Nutritional and Healthy Properties of Foods

Dietary sugars have two sources, either as naturally contained sugar or as added sugar during formulation or processing steps. Correlating the sugar occurrence in foods, either rich or poor in nutrients, with diet quality is challenging in those studies that focus on products with added sugar but do not assess the total sugar, and the food intake is generally concomitant with other foodstuffs that, may or may not, have sugar too [45].

A certain amount of fat in the diet is essential for the development of nervous system tissues, for the assimilation of fat-soluble vitamins and for maintaining an energy balance in healthy individuals. For years, dietary fat was assumed to increase blood cholesterol levels, leading to an elevated risk of cardiovascular diseases. However, current evidence, based on intervention trials and epidemiological studies, does not support the “diet cholesterol—heart disease” hypothesis [52,62,235].

Therefore, edible fats in a balanced diet must be obtained from plant and animal sources. Nevertheless, several studies suggest a relationship between “red” meat intake and illnesses such as cardiovascular diseases, diabetes and cancer [236,237,238]. However, inflammatory processes that trigger these diseases are due to multiple factors such as environmental pollution, lifestyle (stress, sedentarism, and smoking), and unbalanced diet (especially overeating takeaway meals and fast-food consumption). Neutral findings have been obtained as regards the *n*-6 to *n*-3 PUFA ratio and cardiovascular disease protection. The critical aspect is to intake indispensable polyunsaturated fatty acids (*n*3 and *n*-6) in minimal quantities, not considering their relative proportions. This, together with the fact that *n*-3 PUFA and *n*-6 PUFA involve different components, has given rise to the suggestion of the obsolescence of this index [57].

Proteins are considered the sources of amino acids needed to maintain muscle health and, in some way, to have a beneficial role in the prevention of osteoporosis [79,83]. Protein digestion velocity, other macronutrients’ intake and absorption rates would determine the body protein use and therefore the protein requirements of each individual [84]. In this connection, meat intake prevents the loss of muscle mass (i.e., sarcopenia) and promotes bone health since eating foods rich in protein of high biological value stimulates muscle formation joined to physical activity, even if moderate [239,240].

Many edible algae, mushrooms and plants predominately contain an inactive *corrinoid* named pseudovitamin B_12_ [241]. Vitamin B_12_ deficiency is common in approximately 6–12% adults under 60 years old, mainly due to limited dietary intake of animal foods or poor absorption of the vitamin. Vegans as well as other population groups with low intake of animal foods or those with restrictive dietary patterns are at risk of vitamin B_12_ deficiency and are recommended to take supplements [242,243,244,245].

The rising prevalence of vitamin D deficiency (serum 25(OH)D < 50 nmol/L) affects both low- and high-income countries [246]. Very few foods found in nature are good sources of cholecalciferol (animal foods high in fat). Therefore, it is needed to obtain sufficient sunlight for endogenous synthesis of vitamin D, and even then, there will be population groups that do not meet their requirements in sunny regions. The fortification of staple foods provides the majority of vitamin D for those with low sun exposure, low fat intake and plant-based diets. Milk, margarines and cereals are fortified with vitamin D in countries of Northern latitudes [247]. Many types of fermented milks and most alternative vegetable beverages are also enriched with vitamin D but may contain excess sugar and must be evaluated as regards their consideration as healthy food.

Regarding dietary iron, there are two types of compounds, *heme* iron (derived from myoglobin contained in meat and fish) and non-*heme* iron (derived from plant foods, eggs and dairy products). While *heme* iron is well absorbed in the human digestive tract (15–35%), non-*heme* iron is generally poorly absorbed (5–10%), although co-ingestion with vitamin C can improve it [248,249]. Plant-based foods provide non-*heme* iron. Iron intake is higher among adults following plant-based diets, but they have lower iron body stores compared to omnivores [250,251].

The foods richest in zinc are meats from different animals, milk and dairy products and eggs. Several dietary factors can influence zinc absorption. Phytic acid is the main dietary factor known to limit the bioavailability of zinc by binding strongly to zinc in the gastrointestinal tract. This acid is the main phosphorus storage compound found in plant seeds and especially in cereals and legumes, which makes up a high percentage of foods consumed through vegetarian diets and are food staples in developing countries. Diets not rich in animal origin foods must evaluate levels of this essential element to ensure that the required daily intake levels are achieved [252]. Additionally, selenium content in foods and beverages varies geographically between countries. The selenium content of foods of animal origin reflects selenium levels in animal diets, while the selenium content of plants is directly determined by selenium levels found in the soils in which they are grown [253].

As regards sodium as coming from salt intake through food consumption, the spread idea that this must be reduced needs a second thought. The decrease in salt intake offers health benefits when assigned to hypertensive people. However, the same results were not obtained with the normotensive population. Even so, salt reduction can have potential side effects on hormones, lipids, and heart rate of people’s health [89].

In October 2015, the WHO’s International Agency for Research on Cancer [254] classified processed meat as carcinogenic to humans (Group 1) based on sufficient evidence in humans that the consumption of processed meat causes colorectal cancer. Meanwhile, red meat is classified as likely carcinogenic (Group 2A). The state of epidemiological science on red meat consumption and colorectal cancer is characterized by weak associations, heterogeneity, an inability to discriminate the effects of other dietary and lifestyle factors, a lack of dose–response effects, and evidence that weakens over time [255,256]. This can also be applied to many other areas of nutritional epidemiology due to the food intake complexity, with substantial difficulties in isolating the action of single foods or nutrients.

Regarding egg, intervention trials prove that egg intake increases the total cholesterol, LDL-cholesterol and HDL-cholesterol, but not triacylglycerides [68]. This highly accepted idea by consumers is dependent on the plasma reaction to the additional dietary cholesterol provided.

#### 4.1.2. Nutrition Requirements in Different Population Groups

Together with an unhealthy diet, a lack of physical activity is a leading global risk to health. Sustainable physical activities have low environmental impact and are culturally and economically acceptable and accessible [257]. Returning to basic approaches using less equipment and appliances for everyday tasks could contribute to energy balance through increased physical activity and could also decrease resource use. Balancing food intake with energy expenditure will require less food production with accompanying energy savings.

Nutrient intakes should be tailored to meet the needs of different population groups [258]. In this way, due to experiencing a period of rapid growth for infants, this group has more significant nutritional requirements than any other age group and physiological classification. Breast feeding provides ideal nutrition for 4–6 month aged ones, after which a complementary diet is necessary. The high nutritional demand for children and insufficient diets can result in inadequate development. The adolescents’ requirements are like those of adults, with increased intake of protein of animal origin (eggs, meat and fish), consumption of high-energy foods due to increased energy demands, and intake of calcium and iron due to high levels of deficiency [259,260]. Heterogeneous physiological characteristics of elderly people and few studies make it difficult to establish nutritional requirements. Their needs are like those of young adults, with increased vitamin and mineral intake. In the case of pregnant women, almost all nutrients should be increased, especially protein, *n*-3 PUFA, vitamins A and C and folate. Gradual bone loss is common with aging, especially women, irrespective of their ethnicity. Inadequate intake of calcium and vitamin D_3_ increases the osteoporosis risk; thus, 400–800 IU of vitamin D is recommended in menopausal women [261].

#### 4.1.3. Food Classification

The term “meat” is heterogeneously described in regulatory, consumer and scientific environments [135]. Muscle food descriptors and categories are broad and disparately described, which has a big impact on the epidemiological studies’ comparative conclusions. Indeed, the terms “red meat” and “white meat” are not sufficiently informative to precisely classify meat sources. The myoglobin concentration in meat is what defines it as red or white. However, the erroneous myth exist as regards the association of red meat with high levels of saturated fats. As, for example, beef versus veal meats are sometimes considered red and white meat, respectively, yet they come from the same animal. In general, poultry meat is classified as white meat, but turkey legs, although classified as white meat, have a nutritional composition that varies long compared with the breast. These inconsistencies, together with the fact that different parts of the same animal can have different levels of nutrients, may lead to discrepancies in the population estimated intakes [134].

Currently, the classification of food types as “minimally processed”, “processed” and “ultraprocessed” does not properly describe the impact of food processing on health. However, food processing and packaging do have an impact on the environment (see Section 4.2.2). This kind of food classification allows different interpretations. There is not an international definition for unprocessed or minimally processed foods, processed culinary ingredients, processed foods, and ultra-processed foods and drinks (UPFDs), as can be seen in the different NOVA and EPIC (European Prospective Investigation into Cancer and Nutrition) classifications [262]. Nutritional adequacy in industrialized food can be achieved by changing formulations and not the processing levels [263]. Food formulations often increase the energy density of food through “ultraprocessing”, with the addition of added fat, sugar and saturated fats [263]. However, food formulation may also be intended to obtain nutritional benefits. Food processing can lead to improvements in the food security attributes, having a minimum effect on its nutritional attributes. Both fresh and processed foods make up vital parts of the food supply for the consumers, when the aim of processed food is to ensure that sufficient food is available, and the food quality meets human nutrient needs [264].

Since there is no scientific consensus nor legal regulation about the concept of “functional food” [265], it is necessary to clarify and regulate the definition of nutraceuticals and their specific role in the prevention and treatment of pathological conditions, supporting their potential medical use in prevention and therapy only when proven by scientific and clinical data [266]. Therefore, fortified foods are only effective in subjects with special needs [267,268].

### 4.2. Environmental Approaches

As made relevant in Section 3, the environmental impacts of food production and consumption affect air, water, soil, and biodiversity, deriving toxicity for humans, other living beings and the planet. Nevertheless, the high diversity of the studies and research makes it difficult to compare data and extract general conclusions that can guide the definition of environmental sustainability indicators. In this section, some of the most important approaches detected through the reviewed literature that need to be analyzed in depth are discussed.

#### 4.2.1. Holistic Approaches to Environmental Challenges

Both environmental footprint and LCA approaches are useful when analyzing the environmental sustainability of diets. However, in practice, most of the analyzed studies focus on a single or few environmental aspects and hardly any adopt a holistic environmental approach [146,147,152,155,156,163]. Furthermore, these studies do not generally consider aspects such as the rate of local/regional food consumption and seasonality, agrobiodiversity, organic production and consumption or different types of livestock production systems in their approaches, which might be important in avoiding unintended environmental consequences of the recommended dietary shift [6,146,147,155,163,269]. A systems approach integrating the different environmental domains is needed to build resilient food systems [270].

At the European level, a harmonized environmental footprint (EF) methodology is being developed at the product and organization levels based on LCA to quantify environmental impacts [271]. Guidelines are provided to consider different impact-type categories when addressing different product groups and sectors. However, there are some impact categories and aspects not addressed by this method, such as biodiversity, agrobiodiversity and the rate of local/regional foods and seasonality.

At the global level, there are some systemic approaches, commonly used, focusing on the sustainability of food and agriculture systems, such as the SAFA (Sustainability Assessment of Food and Agriculture systems) [272] and the MEMIS framework (Framework for Assessing the Sustainability of Natural Resource Management Systems) [273], which include not only environmental but also socioeconomic attributes and criteria, defined in a case-to-case basis in the latter.

To date, these three methods have not been applied to diets. However, it might be interesting to assess the product options and to explore and adapt their application to diets.

#### 4.2.2. Rate of Local/Regional Food Consumption and Seasonality

“Local/regional food” is produced within a short distance of where it is consumed (up to 100 km or miles) and purchased directly from the producer or with one intermediate between the consumer and producer. Production “in season” refers to the minimum artificial conditions used to grow products, without heated greenhouses in the local agro-environmental conditions and no long-term cold storage [163]. The origins and seasonality of food are important factors to consider in developing sustainable approaches [274,275]. Air-transported and out-of-season produced vegetables and fruits have considerably higher carbon footprints than those produced and consumed locally [276]. Moreover, local products are usually associated with more sustainable agricultural practices [161]. According to the database of the French Environment and Energy Management Agency [277], 1 kg of lettuce produced in a French heated greenhouse emits 11 kg of CO_2eq_, whereas 1 kg of lettuce produced in season generates almost 34 times fewer emissions (0.3 kg of CO_2eq_). Considering that 1 kg of bovine calf produced in a conventional manner emits 6.4 kg CO_2eq_, in this particular case, 1 kg of beef is more environmentally beneficial in terms of GHG emissions than 1 kg of greenhouse-produced lettuce. Nevertheless, the trade-offs between local and seasonal food production need to be analyzed on a case-by-case basis. For instance, Hospido et al. [278] estimate that importing Spanish lettuce to the UK during the winter months results in three to eight times fewer emissions than producing lettuce locally. The same applies for other foods: tomatoes produced in greenhouses in Sweden use 10 times as much energy as tomatoes imported from Southern Europe when in season [279]. Furthermore, the food preservation methods add variability and complexity to the above. Food processing and packaging can cause significant air pollution, water use and, if not properly treated, could be a source of environmental waste production and lead to increasing disposal and pollution problems [280,281]. In summary, local food production is necessary but not sufficient to ensure the best choice from an environmental point of view. Seasonality and preservation methods are also key variables to be considered in the assessment.

#### 4.2.3. Agrobiodiversity and Organic Production and Consumption

Agrobiodiversity, or agricultural biodiversity, encompasses the variety and variability of animals, plants and micro-organisms that are necessary for sustaining key functions of the agro-ecosystem, including its structure and processes for, and in support of, food production and food security [282]. It refers to interactions between agricultural management practices, farmers’ resource endowments, bio-physical resources, and species [283]. Agrobiodiversity conservation and promotion are essential to achieving food security and nutritional, environmental and socioeconomic goals [185,187,188,189,190,191,192,193,194,284,285,286,287]. Some authors have proposed including agrobiodiversity indexes in diet sustainability assessments [187,193] (Table S2). Even if the relationships between agrobiodiversity and human interactions have been clearly identified [194,286], a wide variety of ecosystem services and environmental benefits could be further analyzed and accounted [193].

Today, organic agriculture based on agro-ecological principles is well characterized, controlled, certified and labeled [163]. These food production methods refrain from using synthetic fertilizers and pesticides, GMOs, and intensive animal husbandry and promote crop rotation focused on soil fertility and closed nutrient cycles [163,269]. The feasibility of organic agriculture has been contested at a global scale, as a conversion to organic agriculture implies lower yields and requires more land than conventional agriculture. However, in combination with complementary measures such as reductions in food waste and food-competing animal feed from arable land, with a corresponding reduced production and consumption of animal products, organic agriculture can help provide sufficient food for the population while simultaneously reducing environmental impacts [269].

To sum up, adopting agrobiodiversity approaches and related indexes knowledge and research, as the basis for a healthy environment, would lead the way to develop more comprehensive diet sustainability assessments and ultimately to more sustainable diets.

#### 4.2.4. Livestock Production Systems

Current data show a substantial contribution of the livestock sector to environmental resource use and pollution. It is estimated that this sector is responsible for approximately 13% of global GHG emissions [288,289], for occupying 26% of the total ice-free land surface area (22% through pastures and rangelands and 4% of cropland used for feed) [289,290] and for 29% of the water footprint [291].

However, the different types of livestock production systems, including extensive grassland-based systems, intensive landless systems, and mixed farming systems, vary considerably in terms of environmental pressure [292]. For instance, extensive grassland-based systems and silvopastoral systems with appropriate stoking rates, which generally use land not suitable for other purposes (i.e., there is not feed–food competition), can help store carbon in the soil and lower livestock emissions [293]. The blue water footprint and nitrogen-related grey water footprint are also reduced [294], and the supporting biodiversity and ecosystem services are improved [295,296]. Based on a land use optimization model, Van Kernebeek et al. [297] concluded that moderate meat consumption is more beneficial to the environment than vegan and vegetarian diets. Their results contradict the conclusions of previous LCA studies because the latter did not consider the unsuitability of marginal lands for growing crops, the suitability of animals for human-inedible products and the coproduction of meat and milk [147]. On the other hand, besides being fed by biomass produced from marginal lands, livestock can also take advantage of crop residues and food waste, improving the circularity of the food systems [298].

Context-specific holistic assessments of a harmonized scope are needed to evaluate the trade-offs and win-win solutions that could arise from different animal production practices and systems [6]. These considerations highlight the need to take into account the production model and the role of livestock in the agroecosystems.

### 4.3. Socioeconomic Approaches

#### 4.3.1. Supply

From the supply side, any proposal for a sustainable diet must have its feasibility and scalability evaluated, that is, whether it can be empirically implemented on a large scale to feed the world at affordable prices. Any action to promote more sustainable farming techniques must lower risks for producers of undertaking modes of production that benefit the environment but also guarantee producer’s long-term business survival (especially in low-income countries). In addition, the availability of functioning supply chains requires not only cooperation among supply chain actors (including farmers, producers and other firms) but also reliance on other supporting functions such as transport networks, standards and regulation enforcement, and credit markets.

The sustainability of the food supply chain is affected by several factors, such as (1) the length of food supply chain networks with shorter or larger distances and more or less direct relationships between producers and consumers, (2) the effects of technology improvements in increasing the efficiency of the food supply chain, and (3) possibilities for food supply chain optimization via increased productivity or waste reduction. Additional research efforts to reduce food loss through preservation, reduce transit times and benefit local and seasonal production are important to avoid unintended consequences of recommended dietary shifts [299].

In line with the reviewed literature, the projected increase in the world’s population, coupled with rising incomes, will contribute to an increase in global demand for livestock products to obtain proteins of high biological value [4,20]. In this context, it will be necessary to seek new ways to supply protein from a variety of sources. There is growing research and development focused on alternative protein-based foods using edible insects, algae or cell culture production [219]. Only the creation of new production models based on energy efficiency, the robotization of the agrifood industries and the use of renewable energy could achieve both sustainability and high nutritional value for many of the foods in our diet which need to be provided to the markets at reasonable costs.

#### 4.3.2. Demand

Food demand studies have often been conducted with a focus on diets and populations in developed countries and lower income groups, where evidence also suggests that healthier diets may be costlier than less healthy diets, which, along with knowledge, accessibility and other factors, most likely presents a barrier to the adoption of healthier diets [300,301,302,303,304,305].

Some aspects about the potential for cultural or birthplace bias should be considered. Low-income populations generally cannot afford healthy food and base their diet mainly on vegetables. In relation to plant-based dietary patterns, vegetarian diets show a modest cardiovascular benefit. However, vegetarians are more likely to have lower iron stores than non-vegetarians [251,306]. Vegan mothers present increased risks of delivering newborns with low birth weight than omnivorous mothers [307,308,309,310]. Scientific data do not allow us to draw firm conclusions on the health benefits or risks of present-day vegan diets relating to the nutritional or health status of children and adolescents in industrialized countries [309].

Owing to birthplace bias, rice is a staple food for over half the world’s population, with Asia being a high rice-consuming continent [311]. The contribution of rice to estimated zinc intake is very low coupled with thiamine deficiency. Therefore, this population will need to consume foods rich in this element, such as meat, milk and dairy products, pulses and seafood [312].

Individual preferences, beliefs and cultural traditions are key in shaping food consumption patterns [313]. For instance, insects are part of the food culture in some countries, while in other countries, such as Western countries, populations are reluctant to accept insects as food because they are usually considered pests and sources of contamination [219]. Gaining a deeper understanding of consumers’ attitudes, purchase behavior and buying motives regarding different sustainability attributes is recommended for future studies [147].

Consumers are seeking new foods that offer variety, hedonic experiences, welfare, safety and especially health benefits, but they also consider environmental impacts by selecting foods developed based on concepts of sustainability and the circular economy [314]. In recent years, vegan alternatives have emerged, transforming plants into products named like those used for meat source products but without using animal resources. These “meat analogues” are intended to serve as substitutes for minced meat such as in burgers. These foods are composed of legumes, cereals, spices and food additives to create foods of acceptable organoleptic quality, as shown by an increase in the consumption of such foods [315]. The emergence of new sensitivities related to food and diet configurations has led to the emergence of plant-based diets. However, plant-based diets, when not planned properly, may increase risks of health problems emerging from nutritional deficiencies of minerals such as iron, iodine, and zinc or vitamins A, D, and B_12_ and folate, among others. This has led to the development of novel food products, such as fortified plant foods or “nutritional supplements’” to prevent nutritional deficiencies in the vegan diet.

## 5. Indicators for a Sustainable Healthy Diet

After an exhaustive and critical literature review, the published studies show great heterogeneity in their methods and results. Great advances have been made during the last few years, but, hitherto, they are still insufficient to obtain conclusive results.

Based on the above analysis and reflection process, a comprehensive set of indicators is recommended from environmental, health and socioeconomic perspectives (Table 5). The proposed indicators include not only frequently used indicators such as the carbon footprint, water footprint or land footprint but also other elements generally missing from sustainable healthy diet assessments, such as agrobiodiversity and organic certified food criteria. This harmonized set of indicators could be a step forward for the assessment and comparison of diets in different countries and contexts.

Finally, there are some fields in need of further research in order to arrive at robust scientific conclusions, such as the environmental impacts of the different production systems, including those of alternative proteins, packaging systems or dietary supplements [316,317].

## 6. Conclusions

The recent and growing literature on sustainable healthy diets addresses several aspects related to the economic, social and environmental dimensions of nutrition and health. This body of work covers a wide range of approaches, from LCA to environmental footprint assessment, and countries, mainly developing but also some developed countries. This literature has and will continue to inform policy, business decisions and dietary guidelines with increasing influence.

Feeding the world in a sustainable manner will entail two strands of work. First, it will be important to use a uniform set of parameters and harmonized scopes that properly integrate economic, social, and environmental aspects when defining sustainable and healthy diets in dietary guidelines. This will curtail potential environmental burdens or impacts transferring to other sectors or resources. Second, achieving sustainable diets implies considering culturally sensitive and context-specific approaches using different practices and production systems.

## Figures and Tables

**Table 1:** Specific keywords used in the different domains in the literature review.

Nutrition and Health	Environmental	Socioeconomic
Nutrient requirement Bioavailability Nutritional quality Food intake / food consumption Animal food / plant-based food Processed food Enriched food Dietary supplement Serving size Dietary recommendation Dietary guideline Healthy diet Diet quality/diet quality index Disease risk Sustainable diet	Environmental sustainability Footprint Life cycle analysis Agrobiodiversity Greenhouse gas emissions Water consumption Soil health Biodiversity Energy	Socioeconomic costs Economics Trade Productivity Affordability Consumer preferences Culture

**Table 2:** Accepted nutritional criteria for defining a healthy diet (according to mainstream science) *.

Criteria	Rationale	Relevance and Comments	References
Reduce intake of sugars	Dietary sugars have been linked to dental caries, obesity, and cardiometabolic diseases, including type 2 diabetes (T2DM).	Dietary sugars are not more harmful than excess of dietary energy. However, if the energy is excessive, a higher intake of added sugar (especially from sugar-sweetened beverages) might be associated with poorer diet quality and might increase the risk of caries, overweight, and T2DM.	[42,43,44,45,46]
Reduce intake of saturated fat as much as possible	Cardiovascular diseases (CVDs) have been linked to saturated fat intake based on observational studies.	Cardiovascular diseases (CVDs) have been linked to saturated fat intake based on observational studies with contradictory results. Some studies question further limiting the intake of such fats. Effect of a specific saturated fatty acid should be considered and not “generic saturated fat”. It is the higher intake of *trans*-fatty acids that is associated with greater risk of CVDs in a dose–response fashion.	[47,48,49,50,51,52,53,54]
Reach a low *n*‑6:*n*‑3 ratio	Anti-inflammatory and anti-aggregatory activities are linked to *n*‑3 PUFA. Conversely, the *n*‑6 PUFAs are considered precursors of pro-inflammatory and pro-aggregatory mediators.	The same ratio can be obtained with different individual amounts of *n*-3 and *n-*6 PUFAs. The ratio is about 9.3 when linoleic, arachidonic, a-linolenic, docosahexaenoic, and eicosapentaenoic acids are only considered. This ratio is based on data association and not cause–effect studies.	[55,56,57,58]
Reduce intake of cholesterol	From the 1960s, epidemiological studies have suggested that dietary cholesterol contributes to the increased risk of CVD.	Evidence from observational studies conducted in different countries does not indicate a significant association with cardiovascular disease risk. Findings from intervention trials prove that dietary cholesterol does not increase plasma cholesterol. Likewise, the impact of dietary cholesterol on the immune response remains unclear. Not all subjects respond equally to dietary cholesterol. Dietary guidance focused on dietary patterns is more effective in improving diet quality and promoting cardiovascular health.	[59,60,61,62,63,64,65,66,67,68,69,70]
Protein amount and source	The recommended protein intake level (0.8 g/kg) was derived as a minimum amount to avoid the loss of body nitrogen. Animal proteins have been linked to increased risk of many diseases (cancer, CVD, diabetes, osteoporosis, etc.)	The recommended protein intake level (0.8 g/kg) was derived as a minimum amount to avoid the loss of body nitrogen. Higher protein intake can help maximize health benefits, particularly in older individuals. Amounts of protein above recommended do not appear to have harmful effects. It is unclear whether the relation animal proteins–diseases (cancer, CVD, T2DM, osteoporosis, etc.) is indicative of a causative effect or due to other diet and lifestyle factors. The role of plant or animal proteins in diseases or mortality is difficult to isolate. If there is a difference, it is reduced. Evidence to date is inconclusive. Globally, plant protein consumption is not more advantageous than animal protein consumption and vice versa.	[71,72,73,74,75,76,77,78,79,80,81,82,83,84,85]
Reduce intake of salt	Several dietary guidelines, health organizations and government policies recommend population-wide sodium restriction to prevent hypertension and related comorbidities such as heart failure.	The reduction in blood pressure is clinically relevant in the hypertensive population, especially in the elderly and Black ethnicity populations. There is not enough scientific evidence to recommend salt reduction in the general population. Health policies should focus on the target population.	[86,87,88,89,90]
Intake of dietary fiber	Observational studies suggest a protective role of dietary fiber intake in colon cancer risk.	Colon cancer (CC) is an entity with different molecular subtypes. Epidemiological studies can mask these subtypes. Few studies consider environmental and molecular factors together. Regarding CC patients, increased fiber intake does not reduce the risk of recurrence.	[91,92,93,94,95]
Reduce intake of palm oil (PO)	PO contains a high amount of saturated fat (40–50% of total fat). Their low consumption has been proposed as a policy to reduce deaths due to CVD.	Consumption of PO is associated with an increase in LDL cholesterol, but irrelevant clinically. Insignificant effects on fasting glucose and insulin. The studies to date do not establish strong evidence for or against PO consumption relating to cardiovascular disease risk and cardiovascular disease-specific mortality.	[96,97,98,99,100,101,102]
Reduce intake of dietary fats (butter and margarine)	Butter and margarine contain high amount of saturated fats. Saturated fats have been linked to high CVD risk.	Butter consumption was weakly associated with all-cause mortality in prospective studies. A theoretical analysis suggests that substituting butter with tub margarine may be associated with reduced risk of myocardial infarction. Beef fat was more effective in reducing LDL-cholesterol as compared with butter according to randomized trials. The number of studies remains insufficient to conclude a cause–effect relationship between fats and CVD.	[103,104,105]
Reduce intake of whole dairy products	Saturated fats from whole dairy derivatives have been associated with increased risk of chronic diseases including obesity, metabolic syndrome, T2DM, CVD, osteoporosis, and cancers.	Intake of dairy products was associated with a neutral or reduced risk of T2DM and a reduced risk of CVD, particularly stroke. The evidence suggested a beneficial effect of dairy intake on bone mineral density but no association with risk of bone fracture. Among cancers, dairy intake was inversely associated with CRC, bladder cancer, gastric cancer, and breast cancer, and not associated with risk of pancreatic cancer, ovarian cancer, or lung cancer, while the evidence for prostate cancer risk was inconsistent. Consumption of dairy products was not associated with all-cause mortality.	[106,107,108,109,110,111,112,113,114,115,116,117,118,119,120,121,122]
Reduce or suppress intake of red meat	From the 1970s, epidemiological studies have suggested that cancer and CVD risks are linked to red meat. Saturated fat, *heme* iron, N-nitroso compounds, and sialic acid have been implicated as causes of the increased risk.	After multivariate adjustment for dietary and non-dietary risk factors, total, unprocessed, and processed red meat intake were each associated with a modestly higher risk of CVD. Meats, meat products and meat derivatives are inconsistently classified or misclassified into food groups when dietary questionnaires are applied. Studies examining the relation between the consumption or avoidance of meat and psychological health varied substantially in methodologic rigor, validity of interpretation, and confidence in results. Most studies, and especially the higher quality studies, showed that those who avoided meat consumption had significantly higher rates or risk of depression, anxiety, and/or self-harm behaviors.	[123,124,125,126,127,128,129,130,131,132,133,134,135]
Dietary quality index	An index combining the above criteria would allow the objective assessment of diet quality. Such indices would facilitate the implementation of dietary guidelines.	From the 1990s, 50 more indices have been proposed based on nutrients, foods or combining the two. Most recent proposals include inflammatory or cardiovascular risk biomarkers. The main limitations of these indices include: a non-standard methodology, “a priori” scoring, estimation of nutrient and biocompound intakes by questionnaires, failing to distinguish between some food types, difficulty in establishing a strong dose–effect relationship, inclusion of subrogated biomarkers, exclusion of different ethnic groups and phenotype/genotype profiles, poorly defined “lifestyle”, etc. Some recommendations obtained from these indices are unrealistic since they propose food substitutions that nutritionally are not interchangeable.	[136,137,138,139,140,141,142]

* The details of the nutritional and healthy indicators used to assess a sustainable healthy diet are provided in Table S1.

**Table 3:** Indicators of an environmentally sustainable diet *.

Environmental Concern	Indicators and Definition	Relevance and Comments	References
Biodiversity loss	Biodiversity footprint: biodiversity loss related to products and processes.	Land conversion for crop and animal agriculture is the main driver of habitat loss, which currently continues to be the leading threat to biodiversity. Increasing crop yields, reducing deforestation and reducing meat consumption may be the most effective means to prevent biodiversity loss in future years.	[164,165,166,167,168]
Energy consumption and greenhouse gas (GHG) emissions	Energy use: energy used in the production and/or cooking of a product or process.	Food production, transport and consumption require large inputs of energy that have a significant environmental impact. Energy consumption is commonly linked to GHG emissions, as energy generation methods are some of the main emission sources. Global carbon emissions have increased by nearly 50% since 1990. Food production is one of the main causes of climate change.	[169]
	GHG emissions: release of greenhouse gases into the atmosphere.		[170,171,172,173,174,175,176,177,178,179,180,181]
	Carbon footprint: total GHG emissions caused by a product or process, expressed as carbon dioxide equivalent.		[182]
Food waste	Food waste and losses: decrease in the quantity or quality of food. Daily food waste per capita.	Globally, 30% of food is wasted annually (IPCC, 2019). In fact, avoidable food waste represents the largest fraction of overall food waste. The environmental footprints of an average person’s daily food waste are: 124 g CO_2_ eq., 58 L of freshwater use, 0.36 m^2^ of cropland use, 2.90 g of nitrogen use and 0.48 g of phosphorus use.	[4,154,183,184]
Food and vegetable biodiversity (agrobiodiversity)	Agrobiodiversity: variety and variability of animals, plants and micro-organisms that are necessary for sustaining key functions of the agro-ecosystem. There is currently no agreed, standard way of measuring agrobiodiversity in diets, food production or genetic resources. Indices include the Simpson’s diversity, Shannon’s diversity, and Dietary Species Richness. Beyond conventional measures, the Agrobiodiversity Index (ABD Index) is a method of measuring agrobiodiversity in a consistent, long-term manner to be applied across all pillars of sustainable food systems. The ABD Index assesses diversity in production, food markets, consumption, conservation, and seed systems.	Species richness and diversity scores are usually related to adequate levels of micronutrients and presented as a promising solution for food security issues. Furthermore, maintaining genetic diversity is key for agricultural crops and livestock to be able to adapt—naturally or with human intervention—to future needs and challenges and be resilient to disturbances.	[185,186,187,188,189,190,191,192,193,194]
Land use	Land use change: the acquisition of natural resources for human needs (croplands and pastures), often at the expense of degrading environmental conditions.	According to FAOSTAT, in 2017, 50% of habitable land was used for agricultural purposes, of which 77% was used for animal feed. Land and soil degradation is a global challenge that may contribute to food insecurity, higher food prices and climate change in the near future. Overall, changing land use, high-yield cultivars and meat products are the main triggers of land deterioration.	[195]
	Land use: agricultural land required to produce crops for direct human consumption, as feed and for usage in industry and the energy sector, plus the area needed to produce the commodities’ packaging material.		[196,197]
	Human carrying capacity: persons fed per unit land area.		[198]
	Land footprint: amount of land needed to produce food (grasslands, croplands used to produce feed crops, and croplands used to produce crops for human food).		[199,200]
	Forest cover loss: areas of forest cover removed related to land use changes.		[201]
	Ecological footprint: biologically productive area people use for their consumption and pollution (i.e., crop-, grazing-, forest-, fish-, built-up and carbon-uptake land) to the biologically productive area available within a region or the world.		[202,203]
Pesticide use	Chemical footprint: all chemical substances released into the environment which may ultimately lead to ecotoxicity and human toxicity impacts.	Pesticides used in the agricultural production phase are the main contributors to ecotoxicity and human toxicity episodes. The use of such chemicals is not commonly addressed by sustainability approaches.	[149]
Nitrogen (N) application	N footprint: total amount of N released into the environment during the food chain as emissions of nitrous oxide, nitric oxide, ammonia or molecular nitrogen to the atmosphere, or as nitrate or organic nitrogen to the hydrosphere before the food product is supplied to the consumer. In some studies, it has been considered equal to N use.	A 50% increase in the N and P input to agricultural fields from 2010 levels will be required by 2050. N and P losses, agriculture intensification and dietary choices are responsible for eutrophication in many parts of the world and are endangering many freshwater and coastal ecosystems.	[183,184,204,205]
	N loss: nitrogen losses to the environment from agriculture (croplands and animal manure management).		[206]
Phosphorus (P) application	P footprint: total amount of P released into the environment as a result of food consumption. In some studies, it has been considered equal to P use.		[205,207]
Water use and/or scarcity and pollution	Green water footprint: volume of rainwater consumed during the production process. Blue water footprint: volume of surface and groundwater consumed as a result of the production of a good or service. Grey water footprint: the grey water footprint of a product is an indicator of freshwater pollution that can be associated with the production of a product over its full supply chain.	Agriculture (including irrigation, livestock and aquaculture) accounts for approximately 70% of total freshwater use. Actual population growth and climate change scenarios are substantially increasing levels of water stress globally. Some of the most promising means to improve water use efficiency involve a combination of plant-based dietary choices and reducing food loss and waste.	[184,208,209,210,211,212,213,214]
	Water use efficiency: micronutrient output per liter consumptive water use.		[215]
	Blue water scarcity footprint: equivalent amount of water withdrawn from a waterbody at the global average level of stress.		[157,199,216]
	Green-blue water (GBW) scarcity index: ratio of GBW availability and water resource requirements for producing a country-specific 3000 kcal/cap/day model diet with 20% of the energy from animal products.		[210,211]
	Blue water scarcity: blue WF amounts in relation to local blue water availability.		[213]

* The details of the studies on the environmental sustainability indicators used to assess a sustainable healthy diet are presented in Table S2.

**Table 4:** Socioeconomic indicators for a sustainable healthy diet*.

Criteria	Comments	References
**Supply side indicators**	**Those affecting the production and distribution of food**	
Scalability and feasibility	Many of the assumptions of sustainable diet models are too rigid to resist empirical testing: Perfect substitutability among foods; Perfect substitutability of land for different forms of agrarian production; Constant yield growth rates; Resistance of organic agriculture to pests and climate patterns. A key question for any sustainable diet concerns whether it can be empirically implemented on a large scale.	[218,219]
Value chain approach	Value chains consist of involved actors (including public organizations and private firms) and the sequence of activities performed to bring a product from production to the consumer. Functioning supply chains require not only cooperation among supply chain actors (including farmers and between producers and other firms) but also rely on other supporting functions such as transport networks, standards and regulation enforcement, and credit markets.	[219,220,221,222]
Production costs	Local and organic agriculture is less productive per hectare and more vulnerable to climate patterns and pests. These risks elevate production costs and must be considered for producers to undertake modes of production beneficial for both the environment and producers’ long-term business survival (especially in low-income countries).	[223,224,225,226]
Ethical and societal factors	It is necessary to consider the impact on farmers’ livelihoods, especially for smaller operators and those in underdeveloped economies reliant on livestock production for income and wealth.	[219]
**Demand side indicators**	**Those affecting consumer food choices**	
Availability	The availability of sufficient quantities of food of appropriate quality.	[219]
Resilience (stability)	Locally grown, organic, non-processed food lasts fewer days and must be more often purchased close to the production date. Such limitations must be accounted for to encourage the consumer to undertake dietary changes beneficial for the environment and guaranteeing the supply of food (especially in low-income countries).	[224]
Affordability	A healthy/sustainable diet is more costly than a conventional diet. The environmental costs associated with a conventional diet are not high enough to compensate for the difference.	[156,227,228,229,230]
Acceptability	Beyond costs, consumer preferences are affected by a host of factors such as cultural values, family habits, religious beliefs, physical adaptations including those of digestibility and intolerance (different populations show different degrees of tolerance for certain foods), convenience (time to cook), etc., affecting what is acceptable for different consumers.	[231,232,233]
Access equality	Income inequality increases the likelihood of severe food insecurity. The likelihood of being food insecure is higher for women than men in every continent.	[217]

* The details of the studies on the socioeconomic indicators used to assess a sustainable healthy diet are presented in Table S3.

**Table 5:** Proposed indicators for assessing sustainable healthy diets.

Nutrition and Health Indicators	Environmental Indicators	Socioeconomic Indicators
Nutritional requirements according to age, sex, and ethnicity (genetic profile could be considered) Physical activity/sedentarism prevalence Balance achieved between energy intake from sustainable sources and energy needs Food diversity and properly typified foods (according to composition, formulation and processing) Food rations adjusted to nutrient/energy requirements (serving size according to age and physical activity) Commonly consumed food’s contribution to energy, nutrient and biocompound requirements Diet-related morbidity/mortality prevalence	Carbon footprint (climate change) Water footprint Land footprint, land use Rate of local/regional foods and seasonality Agrobiodiversity Nitrogen footprint Phosphorus footprint Chemical footprint and ecotoxicity Acidification Eutrophication Material footprint (use of fossil fuels, metal ores, minerals, and biotic resources) Biodiversity footprint Ozone depletion Particulate matter (PM_2.5_ and PM_10_ footprint) Human toxicity (cancer and non‑cancer) Ionizing radiation (human health) Photochemical ozone formation (human health)	Availability Resilience (stability) Affordability Acceptability Access equality Scalability and feasibility Production costs Impacts on farmers’ livelihoods

## Supplementary Materials

The following are available online at https://www.mdpi.com/article/10.3390/foods10050999/s1, Table S1: The **n**utritional indicators proposed for defining a healthy diet according to mainstream science, Table S2: The environmental indicators used for assessing a sustainable and healthy diet, Table S3: The socioeconomic indicators used for assessing a sustainable and healthy diet.
