# Supplementary material for: Indicators and Recommendations for Assessing Sustainable Healthy Diets
Source: Foods. 2021 May 2;10(5):999. doi: 10.3390/foods10050999 (PMC8147455; doi:10.3390/foods10050999)
Supplement: Supplementary file 1 [file foods-10-00999-s001.zip › foods-1181337-supplementary.pdf]

Table S1. The **n**utritional indicators proposed for defining a healthy diet according to mainstream science.

Indicator	Relevance and comments	References
Reduce intake of sugars	The findings of a systematic review applied to 9 guidelines on sugar intake do not support the hypothesis that dietary fructose is more detrimental with respect to subclinical inflammation than dietary glucose or sucrose. The studies were heterogeneous and only small numbers of studies providing limited evidence.	[42]
	A meta-analysis was applied to 13 studies evaluating sugars intake and biomarkers of subclinical inflammation. Using GRADE (Grading of Recommendations, Assessment, Development and Evaluations) approach, evidence quality of dietary recommendations for sugar in dietary guidelines was low to very low.	[43]
	Epidemiological studies have shown an association between fructose-containing sugars and weight gain, cardiovascular diseases and diabetes only when restricted to sugar-sweetened beverages and not for sugars from other sources. Sugar-sweetened beverages are a marker of an unhealthy lifestyle (more calories, exercise less, smoke more and have a poor dietary pattern).	[44]
	Negative association between dietary sugar and diet quality is referred to added sugar rather than total sugar. There was substantial variation in features of study quality, including sample size, so the magnitude of the observed effect was generally small and may not be of clinical significance.	[45]
	Substitution of sucrose and fructose with starch yielded lower LDL cholesterol. Insulin resistance and uric acid concentrations were beneficially affected by replacement of fructose with glucose. The findings are limited by the very low to moderate.	[46]
Reduce intake of saturated fat as low possible.	A rigorous and impartial analysis does not show a significant difference in all-cause mortality or CHD mortality, resulting from the dietary fat interventions or epidemiological studies. Evidence from RCT and prospective studies available to date does not support the current dietary fat guidelines. The evidence per se lacks generalisability for population-wide guidelines	[47,48]
	Only one randomised controlled trial (from 34) and three cohort studies (from 13) were found in children and young people, but these confirmed a positive relation between total fat intake and weight gain.	[49],
	Results of the prospective cohort studies pertaining to the effect of SFA consumption on CVD risk are contradictory. Similarly, the recent meta-analyses of clinical trials related to the effects of SFA substitution on CVD risk provided extremely different results, which is related to the application of different inclusion and exclusion criteria. Differences in results of randomised controlled trials may be caused by different methodologies of dietary parameter changes, varying duration of studies, as well as the time at which they were carried out.	[50]
	Higher SFA intake was not associated with higher IHD risks. The lower IHD risk observed did not depend on the substituting macronutrient but appeared to be driven mainly by the sums of butyric through capric acid, the sum of pentadecylic and margaric acid, myristic acid, and SFAs from dairy sources. Residual confounding by cholesterol-lowering therapy and *trans* fat or limited variation in SFA and PUFA intake may explain our findings[51]	[51]
	The intervention group had significant reduction in serum cholesterol compared with controls. No mortality benefit for the intervention group in the full randomized cohort or for any pre-specified subgroup. There was no evidence of benefit in the intervention group for coronary atherosclerosis or myocardial infarcts. The cholesterol lowering interventions showed no evidence of benefit on mortality from coronary heart disease. Available evidence from randomized controlled trials shows that replacement of saturated fat in the diet with linoleic acid effectively lowers serum cholesterol but does not support the hypothesis that this translates to a lower risk of death from coronary heart disease or all causes.	[52]

Indicator	Relevance and comments	References
	Different carbon numbers of SFAs have been shown to have differential effects on the status of metabolic syndrome (MS), implying that SFAs are not homogenous for the effects. The association between the MS risk and SF depends on the chain length.	[53]
	Total fat, SFA, MUFA, and PUFA intake were not associated with the risk of cardiovascular disease. However, we found that higher TFA intake is associated with greater risk of CVDs in a dose-response fashion.	[54]
Reach a low omega 6:3 ratio	The same proportions can be obtained with different amounts of *n*-3 and *n*-6 PUFAs. This ratio is based on data association and not cause-effect studies.	[55]
	The omega-3 Index (EPA + DHA as a percent of total fatty acids) is a better predictor of CVD risk.	[56,57]
	The EPA:AA ratio may be useful for risk stratification of patients with CVD.	[58]
Reduce intake of cholesterol	Humans differ in their response to dietary cholesterol. Many studies have identified hyper- and hyporresponder people. Assuming that approximately 50% of the general population is integrated of hyporresponders, the impact of dietary cholesterol intake on risk for CVDs becomes weaker. A dietary cholesterol recommendation based on genetic profiles would be more appropriate.	[63,64]
	Increased dietary cholesterol intake results in increase in both HDL and LDL cholesterol levels. Therefore, the ratio of HDL/LDL cholesterol remains unchanged. Several studies have suggested considering the HDL/LDL ratio for assessing CAD risk.	[60,61,65]
	Epidemiological studies on dietary cholesterol and CVD were heterogeneous and lacked the methodologic rigor to draw any conclusions regarding the effects of dietary cholesterol on CVD risk.	[59]
	No epidemiological studies or clinical trials corroborate an increased CVD risk from egg cholesterol.	[66–70]
Protein amount and source	Observational and intervention studies have sought to address the question of whether plant and animal protein differ for reducing CVD risk factors. However, evidence to date is inconclusive and is likely to remain so, because it is difficult to isolate the independent effects of specific proteins. The contribution of other components in the plant or animal food source and the background diet are important considerations. Therefore, rather than debating the merits of healthy dietary patterns that are exclusively plant-based or that include animal sources in recommended amounts, the focus should be on improving overall eating patterns to align with dietary guidelines.	[71–75]
	Plant *vs* animal proteins do not seem to differ in their ability to prevent bone loss. Globally, plant protein consumption is not more advantageous than animal protein consumption and *vice versa*.	[76,77]
	Protein intakes above the current RDA may have some beneficial role in preventing hip fractures and bone mass density (BMD) loss. There were no differences between animal or plant proteins, although data in this area were scarce.<br>There is an association between a dietary protein intake above the current RDA of 0.8 g/kg body weight/day and a reduced hip fracture risk in older adults. In addition, positive trends for total hip and femoral neck BMD were found.	[76,79]
	High protein (HP) diets result in higher glomerular filtration rates (CFR); however, when changes in GFR were compared, dietary protein had no effect. Our analysis indicates that HP intakes do not adversely influence kidney function on GFR in healthy adults.	[80]
	High intake of total proteins was associated with a lower risk of mortality from all causes. Intake of plant protein was also associated with a lower risk of mortality from all causes and CVD. Extrapolation of these findings to the worldwide population should be done cautiously because most studies included in the meta-analysis are from Western nations and few studies have been reported from other countries.	[78,81,82]
	The recommended dietary allowance for protein (0.8 g/kg/day) might be inadequate for maintaining muscle health in older adults, probably because of "anabolic resistance" in aged muscle. Higher protein intake (1.1-1.3 g/kg/day) can help maximize health benefits,	[83–85]

Indicator	Relevance and comments	References
	particularly in older individuals. To maximize anabolism, one should consume protein at a target intake of 0.4 g/kg/meal across a minimum of four meals in order to reach a minimum of 1.6 g/kg/day.	
Reduced intake of salt	Guidelines recommend restricting sodium in heart failure (HF) patients. However, these recommendations are based on expert opinion (level C), leading to wide variability in application and lack of consensus among providers pertaining to dietary salt restriction. Considering the available evidence and weighing the documented benefits versus risks, recommendation for lowering salt intake, both for the general population (to reduce the risk of arterial hypertension and subsequent HF) and in patients with established HF, is questionable. The intake of sodium should be individualized, and further studies are needed to clarify this important issue.	[86]
	Observational studies show that sodium intakes below 2,645 and above 4,945 mg are associated with increased mortality. Given that 90% of the worlds' population currently consumes sodium within the optimal range of 2,645–4,945mg, there is no scientific basis for a public health recommendation to alter sodium intake.	[87]
	Sodium reduction from an average high usual sodium intake level (201 mmol/day) to an average level of 66 mmol/day, which is below the recommended upper level of 100 mmol/day (5.8 g salt), resulted in a decrease in SBP/DBP of 1/0 mmHg in white participants with normotension and a decrease in SBP/DBP of 5.5/2.9 mmHg in white participants with hypertension. A few studies showed that these effects in black and Asian populations were greater.	[88]
	A blood pressure (BP) in the highest 25th percentile of the population showed a clinically significant drop in BP with sodium reduction. The policy of lowering dietary sodium intake in the general population may need to be reframed to target patients with hypertension.	[89]
	The reduction of blood pressure is clinically relevant in the hypertensive population, especially in the elderly and Black ethnicity populations. There is not enough scientific evidence to recommend salt reduction in the general population. Health policies should focus on the target population.	[90]
Intake dietary fiber	An epidemiological association with colorectal cancer risk reduction was found by intake of whole grains. It is believed that the protective mechanism of whole grains is mainly explained by dietary fiber, resistant starch and oligosaccharides. However, the evidence for protective effects of total fiber was classified only as suggestive and therefore could not explain the protective effects of whole grains completely.	[92]
	To date, the evidence from randomized controlled trials does not support that increased dietary fiber intake reduces the risk of CRC or reduces the risk of recurrence of adenomatous polyps within a two- to eight-year period compared with control groups.	[94]
	The epidemiological studies analyzing the association between dietary fiber intake and colon cancer in men were fewer compared to women, although the major effect is observed in men compared to women. However, there are still low evidences related to dietary fiber intake and protective effect on colon cancer according to tumor site.	[91]
	Few studies consider environmental (E) and genetic (G) factors together. Thus, studies incorporating accurate assessment of environmental exposures are encouraged not only to identify novel G×E interactions, but also to discover novel risk loci for CRC by characterizing any underlying G×E interactions.	[93]
	CRC is molecularly heterogeneous and complex, with varying tumor biology. CRC is classified into four molecular subtypes clinically. Traditional epidemiological studies may mask true associations between diet, lifestyle and CRC risk.	[95]
Reduce intake of palm oil (PO)	Based on systematic reviews, ESPGHAN conclude that there is insufficient evidence to suggest that PO should be avoided as a source of fat in infant formulas for health reasons. Inclusion of high palmitate fat blend in infant formulas may have short-term effects on stool consistency.	[96]

Indicator	Relevance and comments	References
	Both favorable and unfavorable changes in CHD/CVD risk markers occurred when PO was substituted for the primary dietary fats, whereas only favorable changes occurred when PO was substituted for *trans* fatty acids.	[97]
	PO consumption results in higher LDL cholesterol than do vegetable oils low in saturated fat and higher HDL cholesterol than do *trans* fat–containing oils in humans. The increases of LDL cholesterol and HDL cholesterol were 0.24 mmol/L and 0.02 mmol/L, respectively. However, it should be kept in mind that LDL or HDL are not the only CVD risk factors.	[99]
	During the years of 1980 and 1997, for every additional kilogram of PO consumed per-capita annually, CHD mortality risk was 68 deaths per 100,000 in developing countries and 17 deaths per 100,000 in high-income countries, whereas stroke was associated with 19 deaths per 100,000 and 5.1 deaths per 100,000, respectively.	[98]
	Overall, there are no significant differences in the effects of palm olein intake on lipoprotein biomarkers compared with other dietary oils. However, dietary palm olein was found to have effects comparable to those of other unsaturated dietary oils (monounsaturated fatty acid– and polyunsaturated fatty acid–rich oils) but differed from that of saturated fatty acid–rich oils with respect to the serum lipid profile in healthy adults.	[100]
	PO consumption had no significant effects on blood total cholesterol, LDL-cholesterol, and triglyceride concentrations, relative to the effects of unsaturated fatty acid consumption. Subgroup analyses revealed that PO has a beneficial effect on HDL cholesterol levels when more than 30% of total dietary energy was constituted by fat.	[101]
	Randomized trials show that differences in fasting glucose and insulin were insignificant when compared to other oils. Current evidence on the effects of PO consumption on biomarkers of glucose metabolism is poor and limited to only healthy participants.	[102]
Reduce intake of dietary fats (butter and margarine)	According to the prospective study, theoretical dietary substitution of butter and stick margarine with tub margarine may be associated with reduced risk of myocardial infarction. This study was based on a self-reported questionnaire. Bias due to potential misclassification cannot be reject.	[103]
	A systematic review on butter consumption and diseases could only identify prospective studies. No RCTs were identified. Butter consumption was weakly associated with all-cause mortality, was not significantly associated with any CVD, and was inversely associated with incidence of diabetes. The finding suggests small or neutral overall associations of butter with mortality, CVD, and diabetes. Conclusions do not support a need for major emphasis in dietary guidelines on either increasing or decreasing butter consumption.	[104]
	The effects of different oils/solid fats on blood lipids were assessed by a meta-analysis applied to RCT. The authors conclude that saturated-fat foods should be replaced by unsaturated-fats foods. Some limitations should be considered. Most of the evidence came from indirect comparisons, showing important heterogeneity and inconsistency, and wide imprecision for several comparisons.	[105]
Reduce intake of whole dairy products	Meta-analysis of 31 prospective cohort studies of low/full-fat dairy intake and CVD was conducted. Relative risk below 1.0 were observed for total dairy and cheese intake. Dairy consumption may be associated with reduced risks of CVD, although additional data are needed to examine dose–response patterns.	[106]
	A meta-analysis include 18 prospective studies to investigate dose-response between dairy intake and stroke risk. Risk reductions were maximal around 125 g/day for milk and from 25 g/day onwards for cheese. No associations were found for other dairy products. Results should be cautious due to high heterogeneity observed.	[109]
	A meta-analysis of 15 prospective studies showed a relative risk below 1.0 for CVD and CHD if cheese consumption was high.	[108]
	A total of 29 cohort studies were processed by a meta-analysis to assess the all-cause mortality, CVD and CHD. No associations were found for high-fat/low-fat dairy with the health outcomes of mortality, CHD or CVD.	[111]

Indicator	Relevance and comments	References
	According to 13 prospective studies included in a meta-analysis, high-fat milk was inversely associated with ischemic stroke. However, heterogeneity was observed in analysis.	[114]
	A systematic review of 13 RTC was conducted to examine impact of dairy intake on cardiometabolic markers. The higher intake of dairy can decrease total cholesterol, and increase HDL cholesterol, LDL and triglycerides, although those were small changes. The impact of dairy products on cardiometabolic risk in adults is not totally clear.	[110]
	Meta-analysis from 15 prospective cohort studies show that low-fat dairy and cheese were associated with reduced risk of stroke, high-fat dairy, and butter were not associated with risk of stroke. Stronger association was found for stroke mortality than incidence.	[107]
	According to a meta-analysis from 12 prospective cohort studies, CHD mortality was no associate to intake of high-fat dairy. However, there was a positive association between milk intake and CHD mortality.	[112]
	Ten cohort studies were included in a meta-analysis to detect association between CVD risk and fermented dairy intake. CVD risk was decreased by yogurt and cheese intakes.	[122]
	A meta-analysis from 13 intervention trials using biomarkers of dairy intake (C15:0; C17:0 and *trans*-16 C:1*n*-7) conclude that high-fat dairy intake is not associated with an increased risk of CVD.	[116]
	A lower risk of T2DM was associate to higher levels of same biomarkers by a meta-analysis from 16 trials.	[113]
	A systematic review performed on 27 trials suggest that dairy intake have beneficial effects on inflammatory biomarkers (C--reactive protein, cytokine, TNF-α, tumour necrosis factor, IL-6, and interleukin).	[120]
	Overview was performed on 8 meta-analysis to examine mortality and dairy intake. A relative risk below 1.03 was obtained for high- and low-fat dairy consumption. Dairy intake is not associated with risk of all-cause mortality.	[118]
	Effects of different types of dairy intake on metabolic syndrome (MetS) parameters (abdominal obesity, low HDL-cholesterol, hypertriacylglycerolemia, hyperglycemia, and high blood pressure) in adults were assessed by an analysis of 31 observational studies. A one-serving/d increment of milk was related to a 12 % lower risk of abdominal obesity, and a one-serving/d increment of yogurt was associated with a 16 % lower risk of hyperglycemia.<br>Effects of high- and low-fat dairy intake on MetS in children were assessed by an analysis of 28 observational studies and 1 intervention trial. The data suggests that consumption of high-fat dairy is not associated with an increase in adiposity measures. The research was less conclusive in serum lipids, glucose metabolism and blood pressure.	[115,121]
	A meta-analysis on 11 population-based cohort studies was conducted to examine association between dairy intakes and cancer mortality. Whole milk intake in men contributed to elevated prostate cancer mortality risk.	[117]
	A meta-analysis based in 19 papers was conducted to examine association between milk intakes and cancer mortality. Cancer mortality was not associate to intake of total milk. However, high-fat milk consumption was associated with increased risk of cancer mortality.	[119]
Reduce or suppress intake of red meat	A prospective study was performed to examine the effect of replacing plant foods for red meat on CHD risk. The daily intake of one serving of combined plant proteins (nuts, legumes, and soy) was associated with a lower CHD risk compared with total red meat. Substitution of whole grains and dairy for total red meat was also associated with lower CHD risk. Participants with higher total red meat consumption were more likely to smoke and consume alcohol. They had higher intakes of total energy and *trans* fatty acids but were less physically active.	[123]

Indicator	Relevance and comments	References
	The association between processed meat intake and cancers, T2DM, and CVD was examined by a meta-analysis of 22 systematic reviews. None of the systematic reviews reached the maximum score of quality according to AMSTAR (A Measurement Tool to Assess systematic Reviews). According to GRADE, the quality of evidence was very low for the individual outcomes. The rating was based on observational study design, in combination with serious risk of bias, and/or serious imprecision.	[124]
	An intervention trial investigated the effect on cardiovascular risk factors reducing red and processed meat consumption (≥4 times per week) in healthy, non-obese omnivores. Mean total, LDL and HDL cholesterol were reduced in males. Hemoglobin concentration, plus red and white cell count fell during the intervention.	[126]
	A meta-analysis of 31 prospective studies was applied to evaluate the dose-response between red and processed meat consumption and all-cause mortality, cardiometabolic outcomes (stroke, myocardial infarction, and T2DM). Limitations were observational design, and bias associated with dietary measurement. The certainty of evidence was low to very low for all-cause mortality and CVD. Reducing the red and processed meat intake may result in a decrease in risk for cardiometabolic disease and mortality. The magnitude of absolute effect is very small, and the certainty of evidence is low.	[128]
	A meta-analysis of 42 epidemiological studies examined the association between red and processed meat intake and gastric cancer risk. Risks of highest versus lowest consumption for red meat and processed meat were positive for case-control studies but negative for cohort studies. Subtype of gastric cancer suggested null results for gastric cardia adenocarcinoma and gastric non-cardiac adenocarcinoma.	[130]
	A prospective study in UK conclude that a 70 g/day red meat intake was positively associated with a 32% greater risk of colorectal cancer. A heterogeneity by sex was observed. There was no association in women. Main limitation was the method of dietary assessment: the questionnaire only included a subset of food items that do not constitute the full scope of dietary intake, and therefore total energy intake could not be calculated.	[125]
	A meta-analysis of 105 cohort studies were selected to evaluate the effect of different amounts of red or processed meat, on all-cause mortality, cardiometabolic outcomes, and cancer incidence and mortality. Studies comparing vegetarians with non-vegetarians were also considered. The lowest and highest categories of adherence in red and processed meat were compared. The certainty of evidence was classified using the GRADE criteria. The most common methodological limitations were lack of repeated measurement of intake in the dietary patterns, and the heterogeneity of dietary patterns across studies. Outcomes: 1) low-certainty evidence was found linking dietary patterns lower in red and processed meat intake with small reductions in adverse cardiometabolic and cancer outcomes; 2) the dietary patterns lower in red or processed meat intake are associated with a small reduction in risk for all-cause mortality, cardiometabolic outcomes, and cancer outcomes.; 3) the stronger associations in studies of dietary patterns high in red and processed meat intake suggest the possibility of confounding by other dietary characteristics correlated with red and processed meat consumption.	[129]
	The authors propose replacing the GRADE criteria by the NutriGrade criteria to prove link between red meat intake and harmful outcomes (increased risk of CVD, T2DM and cancer). However, the new criterion has serious limitations because it is not sufficiently justified (e.g., inconsistency of weighting factors). NutriGrade is an *ad hoc* criteria to reinforce the prejudices established in the hypotheses.	[127]
	Meat consumption may be associated with a moderately higher risk of depression. Outcomes were: 1) the substantial level of heterogeneity; 2) the relevant literature was limited, and a small number of studies were included; 3) the food frequency questionnaire, diagnostic criteria of depression and the selection of adjusted factors were not uniform; 4) few studies specified the varieties of meat.	[133]
	The results from our meta-analysis showed a significant association between red and processed meat intake and risk of depression.	[132]

Indicator	Relevance and comments	References
	Most studies, and especially the higher quality studies, showed that those who avoided meat consumption had significantly higher rates or risk of depression, anxiety, and/or self-harm behaviors. There was mixed evidence for temporal relations, but study designs and a lack of rigor precluded inferences of causal relations. Our study does not support meat avoidance as a strategy to benefit psychological health.	[131]
	A narrative review monitors the methods utilize for estimating meat intake in U.S. The methods used to classify meats are not consistent with their composition. Inconsistent categorization of meats products, specifically red and processed meat, in nutrition research can increase bias and cause misleading associations between meat intake and disease risk.<br>A systematic review was applied to 369 articles (97 intervention trials and 282 observational studies) describing meat categories and descriptions. Muscle food categories and descriptions are substantively different within and between experimental and observational studies and do not match regulatory definitions. Food terminologies are away from the originally intended meanings. There is a broad diversity, and misclassification of muscle food categories and descriptions throughout chronic disease literature.	[134,135]
Dietary quality index	Overall, 2 approaches can be distinguished to defining dietary patterns. The *a posteriori* approach is obtained through statistical methods using dietary intake data for the population under consideration. *A posteriori*–defined patterns do not necessarily define the healthiest patterns The *a priori* approach are based on current nutrition knowledge and determine conceptually defined dietary components, which are considered important for the promotion of health. However, the accuracy of an *a priori* index approach is limited by the current level of dietary knowledge regarding the diet–health relationship, as well as uncertainties accompanying the index construction process.	[136]
	Dietary intakes of 11 foods and nutrients were weighted to develop the International Diet-Health Index (IDHI). The aim of IDHI was measure the effects of dietary factors on CMD of 186 countries. The range of IDHI was smaller among women than among men for the overall, beneficial and adverse IDHIs. Women experienced smaller detrimental cardiometabolic effects of diet than men. Risk-reducing dietary factors had the greatest beneficial impact on middle-aged populations and the smallest impact on the elderly, whereas the deleterious impact of risk-increasing dietary factors on cardiometabolic health was greatest among younger age groups and declined with age. No single risk-reducing dietary factor contributed most across countries; however, nuts contributed least to the index in most countries. Among risk-increasing factors, red meat generally contributes the smallest proportion to the IDHI.	[137]
	A systematic review from 64 epidemiological studies was performed to synthesize the association between diet quality and cancer (risk and mortality). Fifty-five different diet quality indices were identified. Results suggest no relationship between diet quality scores and overall cancer risk. No consistent relationships between diet quality scores and cancer mortality were found. The relationship with cancer mortality is not conclusive, suggesting additional factors impact overall cancer survival. Results suggest an inverse associations for diet quality scores and risk of breast, colorectal, head, and neck cancer.	[138]
	A meta-analysis of 27 cohort studies was applied to examine the association between 6 diet quality indices and cancer mortality. Recommended Food Score, Dietary Diversity Score (DDS), and HEI-2005 were not related with cancer mortality. The study was unable to investigate the association for specific types of cancer.	[140]
	Diet of 126 adults was assessed by a 9-item questionnaire (diet risk score, DRS) to estimate cardiometabolic risk. DRS was compared to Healthy Eating Index (HEI-2015). The DRS moderately, inversely correlated with the total HEI-2015 score. There are many limitations in DRS criteria, but the most important is the misclassification of foods. For example, peanut butter and nut butter are included in the group of nuts (added sugar and salt).	[139]
	A meta-analysis of 27 cohort studies was applied to examine the association between 6 diet quality indices and cancer mortality. Recommended Food Score, Dietary Diversity	[140]

Indicator	Relevance and comments	References
	Score (DDS), and HEI-2005 were not related with cancer mortality. The study was unable to investigate the association for specific types of cancer.	
	A critical analysis of 31 diet quality indices was conducted previous classification in: 1) those based on country-specific dietary patterns, 2) those adapted from distinct dietary guidelines, and 3) scores specific to key diet-related factors associated with CVD risk. Scores were obtained from 59 observational studies. Selection and weighting of food groups within a score were considered, since food groups are not necessarily equivalent in their associations with CVD. The lack of absolute cut-off values, emphasis on increasing healthy food without limiting unhealthy food intake, and absence of validation of scores with biomarkers or other objective diet assessment methods further complicate decisions regarding the best indices to use.	[141]
	This paper systematically reviewed the dietary metric (based on micronutrients, foods and food groups) for assessing malnutrition linked to non-communicable diseases (NCDs). Twelve metrics were identified for NCDS. Six dietary metrics used food groups only, and six used foods and nutrients. None of the dietary metrics used only nutrients. Only four NCD dietary metrics had convincing evidence of protective associations, mainly for all-cause mortality, CVD, T2DM, total cancer, and cancer mortality. None of the dietary metrics had been validated against NCD outcomes.	[142]

Table S2. The environmental indicators used for assessing a sustainable and healthy diet.

Indicator	Geographical scale	Main findings	References
	**Global**		
GHG emissions (g CO_2 eq.), freshwater use (l), cropland use (m^2), N and P use (g).	International (125 countries)	This study finds out that the environmental footprint in average person's daily food waste is: 124 g CO_2 eq., 58 l freshwater use, 0.36 m^2 cropland use, 2.90 g nitrogen and 0.48 g phosphorus use. Cereals, meat, and sugar were major food groups contributing to environmental impacts.	[183]
WF and WS	Global scale	In this study authors studied the relationship that exists between the Water Footprint (WF) and the Water Stress (WS) of maize cultivars in different regions of the world. Results showed that the relationship between both indicators it is usually weak, meaning that having only one of them in consideration may not be enough to have an idea of the overall environmental impact. They find out that low pressures were sometimes related with high impacts and counter wise.	[213]
Spiral approach		Authors identified 11 key steps to achieve agro-ecological outcomes based in biodiversity principles. This innovative tool presents the opportunity to perform biodiversity-based farm interventions in order to improve agro-ecological outcomes.	[167]
Climate change, ozone depletion, human toxicity/cancer effects, human toxicity/non-cancer effects, particulate matter, ionizing radiation, photochemical ozone formation, acidification, terrestrial eutrophication, freshwater eutrophication, marine eutrophication, freshwater ecotoxicity, land use, water depletion, resource depletion, land occupation, water consumption, water scarcity footprint	International (21 countries)	This study aimed to analyze the differences between plant-based fats over dairy butter and cream. Authors carried out a LCA (Life-Cycle-Analysis) showing the values for climate change (CO_{2eq}/kg), land occupation (m^2 y/kg), land use (kg C deficit/kg), water resource depletion (m^3 water eq/kg), water consumption (m^3/kg) and water scarcity footprint (m^3 water eq/kg). All plant-based fats showed lower environmental impacts than dairy butter and cream, with a mean of 3.3 kg $CO_{2\ eq}$ for 212 plant-based fats over a 12.1 kg $CO_{2\ eq}$ for 21 dairy butter products.	[148]
GHG emissions ($GtCO_2$-eq y^{-1}), cropland use (M km^2), water use (M km^3), N application (Tg N), P application (Tg N), biodiversity loss.	Global scale	This study considered three different scenarios for assessing the environmental impacts of food production. One of the scenarios was referred to the dietary shift towards more healthy diets. The other two scenarios were referred to technological and management-related changes in food production and food loss and waste. Results showed that shifting to plant-based diets would reduce in an 80% the GHG emissions, reducing food waste would reduce a 14% cropland use and a 33% the biodiversity loss and technological and management improvements would suppose a 75% reduction in biodiversity loss, a 30% reduction in water use and a 26% reduction in P and N application.	[6]
GHG emissions (kg CO_2/kg product),	International. (150 countries)	This study considered a reduction in animal-source foods as the main environmental objective. Results	[160]

Indicator	Geographical scale	Main findings	References
water use (m^3/kg product), cropland use (m^2/kg product), N (kg N/t product) and P (kg P/t product) application.		showed a great difference between low-income countries and upper-middle and high-income countries. In high-income countries GHG emissions were reduced an 84%, N and P application a 38% and a 35% respectively, and cropland use a 29% (when shifting to plant-based diet). However, water consumption increased a 16%.	
Ecosystem status, Per-Capita GHG emissions, Per-Capita blue water consumption, Per-Capita Land Use, Per-Capita non-renewable energy use, Per-Capita Biodiversity footprint.	Global scale. International. (156 countries)	According to this study, 52% of the world's population is exceeding the carbon footprint threshold (~750 kg CO_2 per capita/per year). Data shown in this study demonstrated that bovine meat consumption is the main contributor to GHG emissions (followed by milk, pig meat, poultry and goat meat). The main contributors to water consumption (globally) are rice, wheat and sugar. Those countries sourcing animal food from extensive ruminant production have the highest land footprints. Highest per-capita biodiversity footprints are found in tropical countries due to its species richness per unit area. However, Western Europe and North America have relatively high per-capita biodiversity footprints because of their high imports of food from tropical countries. Finally, shifting towards alternative dietary patterns (vegetarian, vegan…) leaded to high improvements in environmental indicators; mainly because of the reduction in meat consumption.	[154]
Species-area relationship (SAR), Species richness and Evolutionary Loss.	International	Authors expected that future land changes may cause the extinction of 209–818 endemic species in the coming years. Although results showed that hotspots of future biodiversity depends on taxa, the scenario and the metric used, in general terms, a world shifting towards a more sustainable path, including increasing crop yields, reduced meat production, and reduced tropical deforestation appears to be the most effective solution for the current biodiversity loss problem.	[164]
GHG emissions (carbon footprint)	International	This review aimed to analyze the GHG emissions of different international diets such as the Mediterranean, the Nordic or the North-Western diets. Meat and dairy products were the main contributors of GHG emissions. Moreover, the North and Western diets of Europe and the USA appeared to be the least environmentally acceptable diets. The Peruvian, Mediterranean or the Indian diets have the highest sustainability scores; due to the high consumption of vegetables, grains and fruits.	[182]
GHG emissions (CO_2 eq·p^{-1}·d^{-1}), eutrophication (kg PO_3^{-4} eq·p^{-1}·d^{-1}) and land use (ha·p^{-1})	Global scale. (37 countries)	This study aimed to quantify the environmental impact caused by switching from average diets to National Recommended Diets (NRD) in several nations of the world. Results showed that almost all high and middle income countries would be able to reduce 2 times their environmental impact if they followed NRDs. This effect was the contrary for low income countries, where NRDs recommended higher meat consumption due to the protein and micronutrient deficit happening in those countries. Thus, a reduction in meat and dairy	[144]

Indicator	Geographical scale	Main findings	References
		products consumption would be the most effective solution globally.	
Biodiversity loss	Global scale	This study showed the interrelation that actually exists between agriculture and livestock expansion, population growth and biodiversity loss. Many ecosystems of the world are being threatened by food production industry. Since 1980, the main cause of deforestation has been related to crop and ranch construction. Moreover, agriculture is one of the main causalities of GHG emissions to the atmosphere, dead zones, habitat destruction and the consequent biodiversity loss. Actually, 60% of the primate species are threatened to extinction due to habitat loss.	[165]
GHG emissions ($GtCO_{2-eq}$)	Global scale (divided by regions)	This study analyzed the GHG emissions of different dietary patterns. The main findings of this study suggested that meat reduction is the most effective solution to reduce GHG emissions in global meanings. However, authors find out that dietary GHG emissions vary a lot among different regions. In fact, developing countries showed the higher absolute reductions in GHG emissions when shifting to less meat consumption dietary patterns. By the other side, developed countries showed the higher per-capita reduction of GHG emissions when shifting to vegetarian or vegan dietary patterns. In fact, the per-capita average emissions of GHG of a person in a developed country is more than the double (53%) that of a person in a developing country.	[177]
Water footprint	Global scale (divided by regions)	The aim of this study was to observe the combined effects of reducing meat consumption and having less food losses on blue and green water use. Moreover, this study considered the GBW index to estimate the water footprint of each region. Results showed that both actions, reducing animal-based foods consumption and reducing food losses would be effective in a global scenario. Blue water consumption (BWC) would be reduced 11-17% and Green water consumption (GWC) an 18%. However, the combination of both scenarios (animal-based foods were reduced a 25% and half-loss of food was proposed) BWC would be reduced a 23% and GWC a 28%.	[211]
Land use	International	Globally speaking, croplands and pastures have become the most abundant biomes in the world. Since Green Revolution technologies, the use in pesticides (+700%) and irrigated land (+70%) has significantly increased in the last 40 years. Changing land-use practices, salinization and habitat loss are some of the environmental impacts caused by the modern agricultural production and consumption models.	[195]
	**Regional**		
N and P footprints (kg per capita/ per year).	Asia	This study assessed the N and P footprints of Japan, China and India. Results showed a significant increase in both footprints caused by a gradual increase in meat,	[205]

Indicator	Geographical scale	Main findings	References
		vegetable and dairy products consumption (with regional specific variations).	
Climate change, ozone depletion, human toxicity/cancer effects, human toxicity/non-cancer effects, particulate matter, ionizing radiation, photochemical ozone formation, acidification, terrestrial eutrophication, freshwater eutrophication, marine eutrophication, freshwater ecotoxicity, land use, water resource depletion, resource depletion	Europe	The food category with higher environmental impact is meat (beef, pork and poultry). The agricultural phase is the phase with the highest environmental impact due to the high emission rates to the soil, air and water (mainly coming from fertilizers and pesticides). This article highlights the importance of counting food waste and loses in LCA.	[149]
Biodiversity, rate of local/regional food and eco-friendly production and consumption.	Mediterranean region	This paper defines a series of health, nutritional and environmental indicators to assess the sustainability of Mediterranean diets. Authors selected three environmental indicators in order to attain sustainability goals: food biodiversity composition and consumption, rate of local/regional food and seasonality and rate of eco-friendly production and/or consumption. The proposed indicators should be helpful for designing policies in health, education and agricultural sectors.	[163]
DDS (Dietary Diversity Score), DSS (Dietary Serving Score), FVS (Food Variety Score), FGI (Food Group Index).	Developing countries (Asia and Africa)	Diet diversification has shown to be beneficial for health and an affordable, accessible and sustainable strategy to meet wellness standards in developing countries. Authors propose a transition towards traditional food systems to meet sustainability goals.	[284]
N footprint of avoidable food waste (Tg N y^{-1}) and Water footprint of avoidable food waste (km^3 y^{-1}).	Europe (based in 6 national studies: UK, Netherlands, Denmark, Finland, Germany and Romania)	Authors estimated that almost 80% of food waste in Europe is avoidable food waste. Meat is the product group with higher avoidable food waste in both, Nitrogen and Water avoidable food waste footprints.	[184]
Agricultural land use (10^3 ha y^{-1}), energy consumption (TJ y^{-1}), water consumption (Km^3 y^{-1}) greenhouse gas emissions (Gg CO_{2eq} y^{-1}) data from LCA.	Mediterranean Diet Pattern *vs.* Western Diet Pattern. Spain	Animal source food products were the main contributors increasing the environmental footprints of Western and Spanish diets. Data shows that Spanish population would reduce greenhouse gas emissions (72%), agricultural land use (58%), energy consumption (52%) and water consumption (33%) when switching to a Mediterranean Diet Pattern.	[158]
	**National**		
GHG emissions	Sweden	This study carried out in a Swedish school demonstrated that the use of linear optimization can significantly improve the nutritional, environmental and monetary levels of school's lunch menus. In fact, results showed a reduction of a 40% in GHG emissions after diet optimization.	[171]

Indicator	Geographical scale	Main findings	References
GHG emissions, water use and energy use.<br><br>LCA methodology.	Lebanon	The aim of this study was to observe the changes in the environmental footprint of the dietary intake of Lebanese adolescents. This study was carried out using Life-Cycle-Analysis methodology and the three indicators that were taken into consideration were GHG emissions, energy use and water use. Results showed that all the environmental footprints increased between 1997-2009, mainly caused by the significant increase in meat consumption and sugar-processed food and drinks intake.	[161]
Families' contribution to the total species pool (number of species).	India	This study assessed the diversity of wild edible plants in order to ensure food security and nutritional requirements. Many of the wild species that were studied can be easily embedded in the national diet. The incorporation of these diverse and local edible plants can potentially help to achieve sustainability goals, nutritional requirements and affordable and acceptable diets.	[191]
Global warming potential,<br>eutrophication potential,<br>acidification potential,<br>ozone depletion,<br>ecotoxicity,<br>cancer and non-cancer health effects,<br>smog formation potential, and fossil fuel consumption	USA	This paper conducted a tiered hybrid input–output-based life cycle assessment (LCA) to analyse potential environmental impacts associated with current US food consumption patterns and the recommended USDA food consumption patterns.	[147]
Water footprint (l/capita)	Spain	This study compared the Mediterranean diet and current food consumption patterns in Spain from a nutritional and water perspective. The Mediterranean diet is also less caloric, as it contains smaller amounts of proteins and fats and is richer in fiber and micronutrients. Due to the high-embedded water content in animal products, a shift towards a Mediterranean diet would reduce the consumptive WF about 750 l/capita day.	[209]
GHG, water, land and P and N footprints	Switzerland	Different diet scenarios were assessed considering all; health, nutrition, environmental and economic aspects of the sustainability goals. Authors found out that Switzerland's environmental footprint would not exceed the planetary boundaries in three scenarios: RSN (Diet Recommended by Swiss Society of Nutrition), vegetarian and flexetarian diets. In fact, RSN diet appeared to be the most environmentally beneficial diet applied to Switzerland with 54%, 26%, 32%, 33% and 34% reduction in GHG, water, land, nitrogen and phosphorous footprints, respectively.	[156]
GHG emissions	UK	Having in consideration economical, health and environmental aspects, authors developed diets to meet all, income, nutritional and environmental restrictions. Results suggested that it was possible to reduce 57% GHGE meeting all requirements. Broadly, the main changes in all groups were similar: reduce animal-based products and increase plant-based foods.	[176]

Indicator	Geographical scale	Main findings	References
LCA and impact categories (IMPACT 2002+).	The Netherlands (industrial scale)	The aim of this study was to analyze the environmental impact of the industrial production of insect food products as an alternative protein source. Results suggested that plant-based protein sources are still more sustainable than insect-based protein food sources. However, insect-based protein-foods showed to have lower environmental impacts (from 0.33 mPt to 1.82 mPt) than chicken meat (2 mPt). Insect protein production showed to have some environmental benefits in comparison to plant-based protein sources (like water or land use reduction). Moreover, this alternative protein-production system is still considered to be an emerging industry.	[150]
Global warming potential, land use, water depletion, freshwater and marine eutrophication, and particulate matter or respiratory organics	USA	This study analyzed three of the dietary patterns recommended by the Americas Dietary Guidelines 15-20: healthy US-style (US), healthy Mediterranean-style (MED), and healthy vegetarian (VEG) patterns. Several attempts have been performed to include sustainability approaches in those guidelines. In order to assess the environmental impact of these three dietary patterns, authors selected six impact categories of importance (climate change, land use, freshwater eutrophication, water depletion, marine eutrophication and PM. For five of the six impact categories, the VEG pattern showed 42-84% lower environmental impact than the US and MED patterns.	[145]
DDS (Diet Diversity Score), DSR (Dietary Species Richness), MDD (Minimum Dietary Diversity).	Peruvian Andes	Diversification of agricultural products at farm-level was directly linked with more adequate nutrition levels in woman from the Peruvian Andes. This study didn't analyze the environmental benefits obtained from agro-biodiversity.	[187]
Species count on farm (crop+livestock), Production Diversity Score, (HDDS) Household Dietary Diversity Score	Indonesia, Kenya and Uganda	Farm diversification helps to meet micronutrient requirements, increases household incomes, has long-term benefits for the environment, it maintains genetic diversity of species and meets food security requirements. All indexes that have been used in this study are related to nutritional requirements. Further studies should consider studying the linkage between diet diversification and environmental benefits.	[193]
GHG emissions (kg CO_2-eq/kg)	Deutschland	Shifting towards more healthy diets by following national dietary guidelines does not substantially mean a reduction in GHG emissions. In fact, this study demonstrated that diets using low-emission products and reducing meat consumption resulted in a 46% reduction of GHG emissions, whereas adhering current diets to food dietary guidelines didn't showed any significant reduction in GHG emissions. Authors criticize the lack of environmental information included in food-based dietary guidelines.	[178]
Energy consumption (kWh)	UK	Food production, transport and consumption require a large input of energy that causes an important environmental impact. Yearly, in the UK, 10% of the household energy use is related to cooking activities. LCA studies do not usually include the cooking phase. There are several variations and factors determining the energy consumption of the different phases; which	[169]

Indicator	Geographical scale	Main findings	References
		makes difficult to assess the environmental impact of this indicator.	
GHG emissions (kgCO_2e/d)	UK	Reducing meat and dairy consumption is not always feasible for meeting health and environmental targets. This study showed that simply by reducing a 50% the amount of any food they consume is sufficient to follow a healthy and sustainable diet. This study was limited to meet GHG emissions requirements without taking into consideration any other sustainability goals.	[174]
Land use (ha p^{-1} y^{-1})	USA	This study calculated the human carrying capacity for ten diet scenarios (two reference diets and eight "healthy diets"). Values ranged from 0.13 to 1.08 ha p^{-1} y^{-1} across the different diet scenarios. The vegan diet showed lower values than ovo-lacto-vegetarian diets.	[198]
Blue water scarcity footprint, greenhouse gas emissions	UK	The increasing preference for pasta and rice and reduction in household purchases of fresh potatoes in the United Kingdom over the period 1981–2010 has resulted in an increase in blue water scarcity footprint and a transfer of burdens from the United Kingdom to Italy and India, however the increased greenhouse gas emissions associated with rice and pasta has been, more or less, compensated by a reduction in emissions associated with purchases of potatoes.	[157]
Greenhouse gas emissions	Australia	The average dietary GHGe were 18.72 ± 12.06 and 13.73 ± 8.72 kg CO2e/day for male and female adults, respectively. The correlation between total energy and GHGe was $r = 0.54$ ($p < 0.001$). Core foods contributed 68.4% and discretionary foods 29.4%. Within core foods, fresh meat and alternatives (33.9%) was the greatest contributor. The modelling of current dietary patterns showed the contribution of discretionary foods to GHGe was 121% greater in the average diet and 307% greater in the "lower quality, higher GHGe" diet compared to the recommended diet. Reducing discretionary food intake would allow for small increases in emissions from core foods (in particular vegetables, dairy and grains), thereby providing a nutritional benefit at little environmental expense.	[173]
Water footprint (l/capita)	Spain	This paper assessed and compared the water footprint (WF) of two recommended diets (Mediterranean and American), and evaluated the water savings of possible dietary shifts in two countries: Spain and the United States (US). The results show that the American diet has a 29% higher WF in comparison with the Mediterranean, regardless of products' origin. In the US, a shift to a Mediterranean diet would decrease the WF by 1629 l/person/day.	[208]
Global warming potential, acidification eutrophication, land occupation, Total Cumulative Energy Demand, biotic natural resource-depletion species,	France	A diet compliant with nutritional recommendations for macronutrients had fewer environmental impacts than the current average French diet. Moving from an omnivorous to a vegetarian diet further reduced environmental impacts. Increasing the *n*-3 PUFA contents in animal rations increased eicosapentaenoic acid (EPA) and docosahexaenoic acid (DHA) in animal food products. Providing these enriched animal foods	[146]

Indicator	Geographical scale	Main findings	References
biotic natural resource-depletion ecosystems		in human diets increased their EPA and DHA contents without affecting their environmental impacts. However, in diets that did not contain fish, EPA and DHA contents were well below the levels recommended by health authorities, despite the inclusion of animal products enriched in EPA and DHA. Reducing meat consumption and avoidable waste at home are two main avenues for reducing environmental impacts of diets.	
Blue water scarcity footprint	UK	The water required to produce the food consumed by the UK was estimated at 52.6 Gm^3/y of which 93% is from rainfall at the point where it falls and 7% is "blue" water withdrawn from surface and ground water resources. Five alternative healthier diets were considered and the impact on the blue water scarcity footprint was modest (ranging from −3% to +2% compared to baseline). However more significant impacts were projected on the geographical distribution of the blue water scarcity footprint. This study has shown that if current trade patterns continue, policies to promote healthier eating in the UK may contribute to increased blue water scarcity at home and in other parts of the world.	[216]
Greenhouse gas emissions	Australia	The GHGe of the average Australian diet was 14.5 kg carbon dioxide equivalents (CO2e) per person per day. The recommended dietary patterns in the Australian Dietary Guidelines are nutrient rich and have the lowest GHGe (~25% lower than the average diet). Food groups that made the greatest contribution to diet-related GHGe were red meat (8.0 kg CO_2e per person per day) and energy-dense, nutrient poor "non-core" foods (3.9 kg CO_2e). Non-core foods accounted for 27% of the diet-related emissions. A reduction in non-core foods and consuming the recommended serves of core foods are strategies which may achieve benefits for population health and the environment.	[172]
Land use (km^2 and m^2 p^{-1} a^{-1})	Germany	This study analyzed different dietary scenario's land use in Germany. The total area used for human consumption in 2006 in Germany was 194,600 km^2. Results showed that shifting to an ovo-lacto-vegetarian or vegan diet and following the national recommendations would lead to a 14% reduction in the total land demand. The main contributors to land use were animal products (meat and dairy products).	[196]
Greenhouse gas emissions (GHGE)	France	High-nutritional-quality diets contained more plant-based foods, notably fruit and vegetables, and fewer sweets and salted snacks than did low-quality diets. After adjustment for age, sex, and energy intake, the consumption of sweets and salted snacks was negatively correlated with diet-related GHGEs, whereas the consumption of animal products and of fruit and vegetables was positively associated with them. After adjustment for energy intake, high-nutritional-quality diets had significantly higher GHGEs (+9% and +22% for men and women, respectively) than did low-nutritional-quality diets.	[180]

Indicator	Geographical scale	Main findings	References
	France	The mean diet-associated GHGE was 4170 g CO_2e/day and a high inter-individual variability was observed. When the total caloric intakes were reduced to meet the individual energy needs, the diet-associated GHGE decreased by either 10.7% or 2.4%, depending on the assumption made on the average physical activity level of the population. The meat and deli meat food group represented the strongest diet-associated GHGE contributor, but the impact of different meat reduction scenarios was modest. In particular, when fruit and vegetables were iso-calorically substituted for meat, either null or even positive diet-associated GHGE variations were observed because the needed amounts of fruit and vegetables to maintain the caloric content of the diet were high. Therefore, substituting fruit and vegetables for meat (especially deli meat) may be desirable for health but is not necessarily the best approach to decreasing diet-associated GHGE.	[179]
	UK	Habitual red and processed meat intakes were 2.5 times higher in the top compared with the bottom fifth of consumers. Under the counterfactual, statistically significant reductions in population aggregate risks ranged from 3.2% (95% CI 1.9 to 4.7) for diabetes in women to 12.2% (6.4 to 18.0) for colorectal cancer in men, with those moving from the highest to lowest consumption levels gaining about twice these averages. The expected reduction in GHG emissions was 0.45 tons CO_2 equivalent/person/year, about 3% of the current total, giving a reduction across the UK population of 27.8 million tons/year.	[170]

Table S3. The socioeconomic indicators used for assessing a sustainable and healthy diet.

Indicator	Geographical scale	Main findings	References
	**Global**		
Equity of land requirements across continents	Global	The paper quantifies the changes in the global agricultural land footprint if the world were to adhere to the dietary guidelines put forth by the United States Department of Agriculture (USDA), while accounting for the land use change incurred by import/export required to meet those guidelines. We analyze data at country, continental, and global levels. USDA guidelines are viewed as an improvement on the current land-intensive diet of the average American, but despite this our results show that global adherence to the guidelines would require 1 gigahectare of additional land—roughly the size of Canada—under current agricultural practice. The results also show a strong divide between Eastern and Western hemispheres, with many Western hemisphere countries showing net land sparing under a USDA guideline diet, while many Eastern hemisphere countries show net land use increase under a USDA guideline diet. We conclude that national dietary guidelines should be developed using not just health but also global land use and equity as criteria. Because global lands are a limited resource, national dietary guidelines also need to be coordinated internationally, in much the same way greenhouse gas emissions are increasingly coordinated.	[197]
Critical criteria assumed:<br>• land currently used for cereal, sugar and oil production can be switched to fruit, vegetable and protein crop production.<br>• crop yields will continue to increase by approximately 1% per year on average	Global	Results show that the global agricultural system currently overproduces grains, fats, and sugars while production of fruits and vegetables and protein is not sufficient to meet the nutritional needs of the current population. Correcting this imbalance could reduce the amount of arable land used by agriculture by 51 million ha globally but would increase total land used for agriculture by 407 million ha and increase greenhouse gas emissions. For a growing population, our calculations suggest that the only way to eat a nutritionally balanced diet, save land and reduce greenhouse gas emissions is to consume and produce more fruits and vegetables as well as transition to diets higher in plant-based protein.	[218]
New value chains<br>production costs<br>food safety<br>scalability<br>consumer acceptance	Global	This paper outlines some potential demand scenarios and provides an overview of selected existing and novel protein sources in terms of their potential to sustainably deliver protein for the future, considering drivers and challenges relating to nutritional, environmental, and technological and market/consumer domains. It concludes that different factors influence the potential of existing and novel sources.	[219]
Demand side policies	Global	This report assesses the availability of demand-side policies and measures, and looks at evidence of these measures' impacts on behavior that directly results in emissions from the agri-food sector. Often discussed demand-side measures include 'soft' measures (e.g. health promotion initiatives, product labeling) and 'hard' measures (e.g. consumption taxes or subsidies). We review here the effectiveness of these measures for dietary change and reductions in food loss and waste, with a focus on developing countries, where agrifood emissions are projected to grow most rapidly and where the gaps in knowledge are largest.	[222]

Shape of the value chain (fragmented vs Concentrated)	Global	This paper uses a global value chain (GVC) approach to bring industrial structure back in the discussion of food standards and smallholders. It proposes a framework to investigate how value chain structure affects small-scale producers through the food standards imposed on them. Different value chain structures generate a welter of food standards and potentially divergent outcomes in the well-being of smallholders. Specifically, the type of lead firm and the degree of market concentration in a given chain differentiate the chain actor's incentives and capacity for adopting and implementing enhanced standards. Value chain structure and food standards shape the conditions of smallholders involved in the chain, offering them different options as they face growing burdens in complying with higher requirements.	[220]
	**National**		
Daily food expenditure<br><br>*Note: it is the only economic parameter observed vs 3 nutritional and 5 environmental parameters.*	Switzerland	Results show that achieving a sustainable diet would entail a high reduction in the intake of meat and vegetable oils and a moderate reduction in cereals, roots and fish products and at the same time increased intake of legumes, nuts, seeds, fruits and vegetables. We identify several current data and research gaps that need to be filled in order to get more accurate results. Overall, our analysis underscores the need to consider multiple indicators while assessing the dietary sustainability and provides a template to conduct such studies in other countries and settings. Future efforts should focus on assessing the potential of different interventions and policies that can help transition the population from current to sustainable dietary patterns.	[156]
	Spain	Objective To evaluate the sustainability of the dietary patterns, according to their effects on health and environment and their affordability. Information from participants is collected every 2 years by validated questionnaires. We assessed three dietary patterns (the Mediterranean, the Western and the Provegetarian dietary patterns).	[228]
Tradition, customs, and lifestyle	Hungary	The study found that dietary choices are complex decisions that have a significant environmental and social impact, but we need to add that thanks to the strong cultural background, the students can keep their sustainable eating and community values abroad, which can also strongly influence the development of the local food supply practices.	[232]
Average cost of food per person within each household<br><br>*Note: the study removed households who purchased a lower than feasible amount of food to maintain the Basal Metabolic Rate of the members of the household.*	UK	The analysis of purchased food items of households were disaggregated into their components with traceable environmental impact data. A relatively low-cost high-quality sustainable diet was purchased by a very small proportion of survey participants showing that there is potential for a carefully chosen diet to be both affordable, sustainable and healthy. But a very small proportion of the UK population purchase a diet that is likely to be compatible with sustaining their own health or that of the planet.	[15]
Cultural, social and personal values around eating	Scotland	The aim of this study was to explore public awareness of the environmental impact of food and their willingness to reduce meat consumption. Twelve focus groups and four individual interviews were conducted with adults from a range of socio-economic groups living in both rural and urban settings in	[313]

		Scotland. Three dominant themes emerged: a lack of awareness of the association between meat consumption and climate change, perceptions of personal meat consumption playing a minimal role in the global context of climate change, and resistance to the idea of reducing personal meat consumption. People associated eating meat with pleasure, and described social, personal and cultural values around eating meat.	
Each individual's diet was cost per day (£/d)	UK	The Dietary Approaches to Stop Hypertension (DASH) diet is a proven way to prevent and control hypertension and other chronic disease. Because the DASH diet emphasizes plant-based foods, including vegetables and grains, adhering to this diet might also bring about environmental benefits, including lower associated production of greenhouse gases (GHGs). Promoting wider uptake of the DASH diet in the United Kingdom may improve population health and reduce diet-related GHGs. However, to make the DASH diet more accessible, food affordability, particularly for lower income groups, will have to be addressed.	[301]
Relative prices of healthy vs unhealthy baskets. Cross elasticities.	Brazil, China, South Korea, Mexico, UK and USA	In high income countries over the last 30 years it seems that the cost of healthy items in the diet has risen more than that of less healthy options, thereby encouraging diets that lead to excess weight. It seems the same may apply in emerging economies, such as Brazil, China, Korea and Mexico, where prices of fruit and vegetables have been rising more than most other foods, including energy-dense processed foods. A strong case emerges for using taxes and subsidies to offset these changes to encourage more consumption of healthy foods and less of unhealthy items.	[302]
Relative price of two baskets was recorded in five neighbourhoods, and the affordability of the baskets was determined across household income quintiles.	Australia	The cost of the healthy and sustainable (H&S) basket was more than the typical basket in all five socioeconomic neighborhoods, with most disadvantaged neighborhood spending proportionately more (30%) to buy the H&S basket. Within household income levels, the greatest inequity was found in the middle-income neighborhood, showing that households in the lowest income quintile would have to spend up to 48% of their weekly income to buy the H&S basket, while households in the highest income quintile would have to spend significantly less of their weekly income (9%).	[303]
Weekly household household food expenditure	Italy	The costs (Euro) per person of the Mediterranean diet (MD) and of the current Italian household food expenditure were considered on a weekly basis according to the 2013 data from the Observatory prices and tariffs of the Ministry of Economic Development and the service SMS consumers of the Ministry of Agriculture, Food and Forestry. The MD resulted to produce a lower environmental impact than the current food consumption of the Italian population. The monthly expenditure of the MD is slightly higher in the overall budget compared to the current expenditure allocated to food by the Italian population, but there is a substantial difference in the distribution of budget according to the different food groups.	[304]
Local and seasonal food is a high-risk strategy for both producers and consumers because crop	UK	The aim of the present paper is to consider the implications of eating seasonal food on the different elements of sustainability (i.e. health, economics, society), not just the environment. Seasonality can be defined as either globally seasonal (i.e. produced in the natural production season but consumed	[224]

production is vulnerable to the climate<br><br>Time-is-money it takes a lot more time to feed oneself in local grown food.		anywhere in the world) or locally seasonal (i.e. produced in the natural production season and consumed within the same climatic zone). Greenhouse gas emissions of globally seasonal food are not necessarily higher than food produced locally as it depends more on the production system used than transportation.<br>The 100 mile diet was started by a couple who wanted to see if it was possible over 1 year to eat only locally produced food, all foods not just fruit and vegetables, within a 100 mile radius of Vancouver city. While they did achieve it, they described the time needed to acquire and prepare food was often the equivalent to a part-time job and achieving a varied and balanced diet throughout the year was challenging, even with preserving foods for winter months.	
Monetization of agricultural production environmental costs (environmental externalities, no actual purchasing costs)	Denmark	Reducing the content of meat and excluding most long-distance imports were of substantial environmental and socioeconomic advantage to the New Nordic Diet when compared with the Average Danish Diet, whereas including high amounts of organic produce was a disadvantage.	[229]
Diet cost	France	The diet-related greenhouse gas emissions for self-selected diets of 1918 adults participating in the cross-sectional French national dietary survey Individual and National Survey on Food Consumption (INCA2) were estimated. "Lower-Carbon," "Higher-Quality," and "More Sustainable" diets were defined as having GHG lower than the overall median value, a probability of adequate nutrition intake (PANDiet) score (a measure of the overall nutritional adequacy of a diet) higher than the overall median value, and a combination of both criteria, respectively. Diet cost, as a proxy for affordability, and energy density were also assessed.<br>A reduction in diet-related GHG by 20% while maintaining high nutritional quality seems realistic. This goal could be achieved at no extra cost by reducing energy intake and energy density and increasing the share of plant-based products.	[175]
Degree of self-sufficiency<br>Total 'virtual' land use – i.e. the land embodied in the food imports to the UK –	UK	Preliminary findings show that the average land requirement for the UK food supply equals 2,588 m2 /capita/year. More than 70% of these land requirements are related to the consumption of animal products, particularly ruminants (33%). Total 'virtual' land use – i.e. the land embodied in the imports to the UK – equals 2·6 million hectare. Considering the total arable area available in the UK, this implies that the total imported area is 45% of total arable area in the UK.	[305]
Relative prices of healthy vs unhealthy baskets.	UK	For many obese children, eating healthily would not necessarily incur prohibitive, additional financial cost, although a poor diet at a budget supermarket remains the cheapest of all options. Cost is a possible barrier to healthy eating for the most economically disadvantaged.	[227]
